# The Oxford study of Calcium channel Antagonism, Cognition, Mood instability and Sleep (OxCaMS): study protocol for a randomised controlled, experimental medicine study

**DOI:** 10.1186/s13063-019-3175-0

**Published:** 2019-02-12

**Authors:** Lauren Z. Atkinson, Lucy Colbourne, Alexander Smith, Catherine H. Harmer, Anna C. Nobre, Jennifer Rendell, Helen Jones, Christopher Hinds, Arne Mould, Elizabeth M. Tunbridge, Andrea Cipriani, John R. Geddes, Kate E. A. Saunders, Paul J. Harrison

**Affiliations:** 1 Department of Psychiatry, University of Oxford, Warneford Hospital, Oxford, OX3 7JX UK; 2 Oxford Health Foundation NHS Trust, Warneford Hospital, Oxford, UK; 3 Oxford Centre for Human Brain Activity, Wellcome Centre for Integrative Neuroimaging, Warneford Hospital, Oxford, UK; 4 Department of Experimental Psychology, University of Oxford, New Radcliffe House, Oxford, UK; 5 Big Data Institute, University of Oxford, Old Road Campus, Oxford, UK

**Keywords:** Bipolar disorder, Calcium blockers, Calcium channel antagonists, Depression, Functional magnetic resonance imaging, Magnetoencephalography, Mood instability, Sleep, Working memory

## Abstract

**Background:**

The discovery that voltage-gated calcium channel genes such as *CACNA1C* are part of the aetiology of psychiatric disorders has rekindled interest in the therapeutic potential of L-type calcium channel (LTCC) antagonists. These drugs, licensed to treat hypertension and angina, have previously been used in bipolar disorder, but without clear results. Neither is much known about the broader effects of these drugs on the brain and behaviour.

**Methods:**

The Oxford study of Calcium channel Antagonism, Cognition, Mood instability and Sleep (OxCaMS) is a high-intensity randomised, double-blind, placebo-controlled experimental medicine study on the effect of the LTCC antagonist nicardipine in healthy young adults with mood instability. An array of cognitive, psychiatric, circadian, physiological, biochemical and neuroimaging (functional magnetic resonance imaging and magnetoencephalography) parameters are measured during a 4-week period, with randomisation to drug or placebo on day 14. We are interested in whether nicardipine affects the stability of these measures, as well as its overall effects. Participants are genotyped for the *CACNA1C* risk polymorphism rs1006737.

**Discussion:**

The results will clarify the potential of LTCC antagonists for repurposing or modification for use in psychiatric disorders in which cognition, mood and sleep are affected.

**Trial registration:**

ISRCTN, ISRCTN33631053. Retrospectively registered on 8 June 2018 (applied 17 May 2018).

**Electronic supplementary material:**

The online version of this article (10.1186/s13063-019-3175-0) contains supplementary material, which is available to authorized users.

## Background

L-type (voltage-gated) calcium channel (LTCC) antagonists (also called calcium blockers or calcium antagonists) such as verapamil and nifedipine are licensed and widely used to treat hypertension and angina. They have also been used sporadically in bipolar disorder and related conditions for more than 25 years [1, 2]. The rationale was provided by diverse reports of altered calcium indices in patients with bipolar disorder and the fact that lithium carbonate, the standard treatment for the disorder, also affects calcium signalling [3, 4]. A recent systematic review concluded that there is no good evidence for — or against — the efficacy of LTCC antagonists in bipolar disorder because there are virtually no controlled trials. Most of the data are observational or uncontrolled, limited to the manic phase of the illness and include results for drugs with uncertain or limited blood-brain barrier penetration [5].

Over the past decade strong evidence has emerged that LTCC genes, especially *CACNA1C* (which encodes the L-type Ca_V_1.2α subunit), contribute to the aetiology of bipolar disorder and other psychiatric disorders as well as to phenotypes which are affected in these conditions such as memory and circadian rhythms [6, 7]. These genomic findings have given new impetus to the study of LTCC antagonists as potential treatments for neuropsychiatric disorders [8]. Whilst the existing drugs are unlikely to be suitable for repurposing in this way, evidence that they can produce ‘psychiatric’ effects would provide an incentive to develop more selective drugs. The latter is a feasible goal because of the differential expression of individual LTCC genes and their isoforms in brain compared to heart and vasculature ([9] and Clark et al., *bioRxiv* 2018: 260562). At present there is virtually no information regarding behavioural or cognitive effects of current LTCC antagonists. Low-quality evidence has demonstrated potential beneficial effects on cerebrovascular cognitive impairment [10, 11]; an electronic health records study has suggested differential admission rates for persons taking LTCC antagonists for depression compared to people taking other antihypertensives [12], and some rodent studies have indicated improved performance on cognitive tasks after administration of LTCC antagonists [13–16].

Here we report an exploratory experimental medicine study of the LTCC antagonist nicardipine given to participants with high mood instability: OxCaMS (Oxford study of Calcium channel Antagonism, Cognition, Mood instability and Sleep). Mood instability was chosen as an inclusion criterion because it is a core feature of bipolar disorder and other disorders with which LTCCs are genetically associated [17]. In addition, it is present in a proportion of the general population (~ 14%) and correlated with poorer cognitive performance and with adverse health outcomes [17–19].

In brief, during a 14-day run-in phase, participants complete repeated assessments of cognition, mood and sleep, and undergo ambulatory electrocardiography (ECG) and actigraphy, functional magnetic resonance imaging (fMRI) of the brain and magnetoencephalography (MEG), as well as measurement of leukocyte LTCC gene expression and calcium flux. After 14 days, participants are randomised to nicardipine sustained release (SR) 30 mg twice a day, or matched placebo, for another 14 days. During this period, all assessments and scans are repeated. The design allows for both between- and within-participant analyses. The overall goal is to determine the effects of LTCC antagonism on behaviour, mood instability, sleep, neural activity and calcium transport, as well as to assess target engagement of LTCCs in the brain.

## Methods/design

**Fig. 1:**
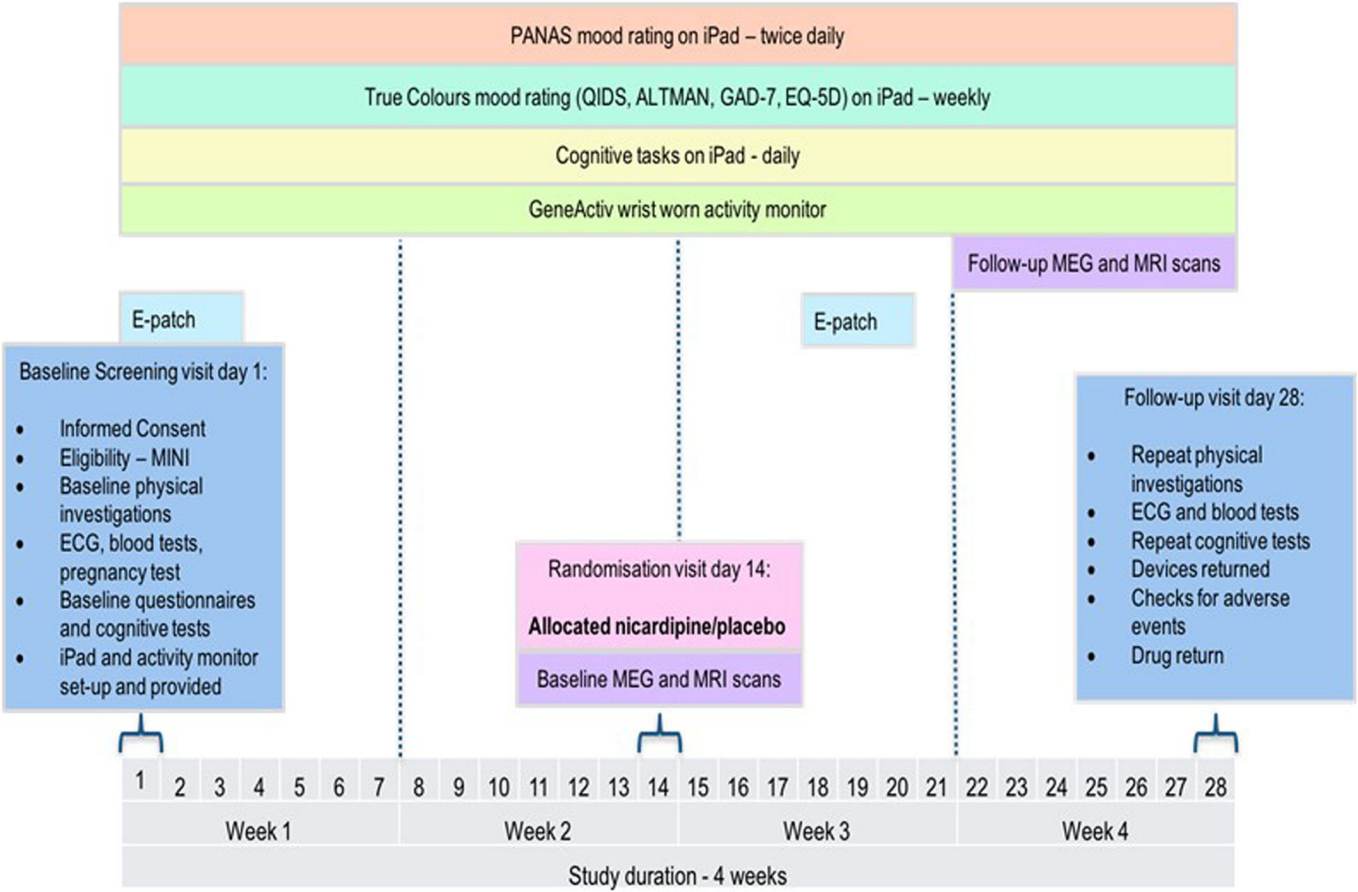
OxCaMS workflow

**Fig. 2:**
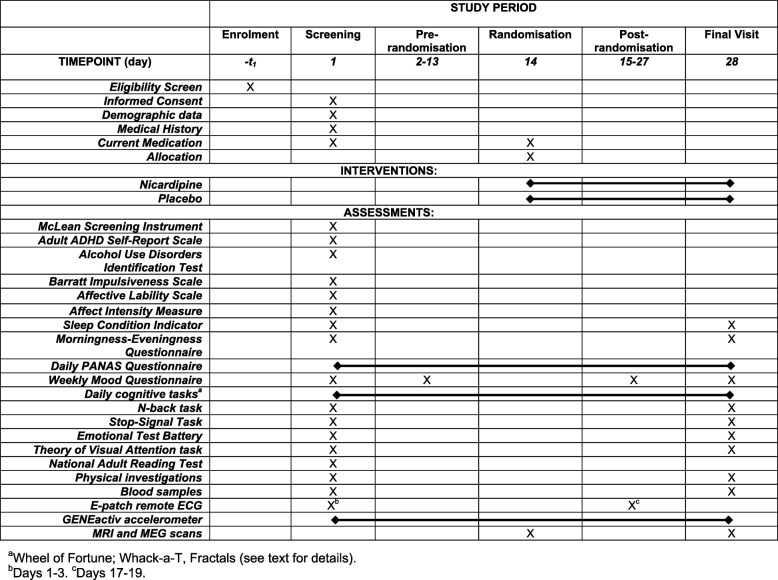
SPIRIT schedule

The OxCaMS trial registration data are listed in Table 1, with an overview of the workflow shown in Fig. 1. The schedule of enrolment, interventions and assessments is shown in Fig. 2. The Standard Protocol Items: Recommendations for Interventional Trials (SPIRIT) checklist is Additional file 1; the current approved protocol (version 1.5) is Additional file 2. The approval letter is Additional file 3.

**Table 1:** Summary of OxCaMS trial registration data

Data category	Information
Registry and ID	ISRCTN33631053
Date of registration	8 June 2018
Secondary identifying numbers	IRAS 213212
Sources of financial or material support	Wellcome Trust, NIHR Oxford Health Biomedical Research Centre, NIHR Oxford cognitive health Clinical Research Facility
Sponsor	University of Oxford
Contact for public queries	PJH (paul.harrison@psych.ox.ac.uk) or KEAS (kate.saunders@psych.ox.ac.uk)
Contact for scientific queries	PJH (paul.harrison@psych.ox.ac.uk) or KEAS (kate.saunders@psych.ox.ac.uk)
Public title	OxCaMS
Scientific title	Oxford study of Calcium channel Antagonism, Cognition, Mood instability and Sleep
Countries of recruitment	UK
Health condition studied	Mood instability
Intervention	Nicardipine sustained release (30 mg bd) or placebo, for 14 days
Key inclusion criteria	Score > 7 on Mood Disorder Questionnaire with evidence of functional impairment. Aged 18–35 years
Key exclusion criteria	Current psychotropic medication. Need for urgent psychiatric treatment. Psychiatric disorder or substance misuse which, in the opinion of the investigator, could compromise safety or data quality
Study type	Primary purpose: experimental medicine, exploratory study Allocation: randomised, double-blind, parallel group. 14 day run-in before allocation
Date of first enrolment	December 2017
Target sample size	40
Recruitment status	Recruiting
Primary outcome	Cognitive variability
Key secondary outcomes	Mood variability, behavioural variability, BOLD and MEG signals at rest and during task performance, sleep parameters, calcium channels in leukocytes

OxCaMS is a 4-week study, with a 2-week pre-randomisation phase, during which time a range of assessments are carried out, followed by randomisation and another 2-week period when the same assessments are continued or repeated whilst the participant takes the drug or placebo. The target recruitment is 40 participants.

Study visits take place at the National Institute for Health Research (NIHR) Oxford cognitive health Clinical Research Facility, with neuroimaging at the adjacent Oxford Centre for Human Brain Activity, part of the Wellcome Centre for Integrative Neuroimaging. Biochemical and genetic assays will be performed within the NIHR Oxford Health Biomedical Research Centre.

Randomisation utilises a computer-generated schedule implemented by a trial manager, who is based at the University of Oxford Department of Psychiatry and is external to the study. The randomisation algorithm minimises on gender, in a 1:1 allocation to either nicardipine SR or matched placebo. Dispensing is by Clinical Research Facility staff.

### Choice of drug and dosing regimen

Nicardipine is a dihydropyridine LTCC antagonist, structurally similar to nifedipine [20]. It has strong evidence for good brain penetrability [21, 22] and occupancy of central LTCCs after repeat dosing [21]. Nicardipine has reported beneficial effects in pre-clinical models of cerebral ischaemia [23], seizures [24] and brain ageing [25]. Nicardipine is a potent blocker of Ca_V_1.2 channels [26]. It is available in a sustained release formulation, allowing twice daily dosing, resulting in easier administration and a lower maximal concentration (C_max_) which decreases the side effects associated with vasodilatation (e.g. flushing, headache). In a study of normotensive young adults, the investigational regimen used in OxCaMS (nicardipine SR, 30 mg twice daily) was well tolerated and produced no significant effects on blood pressure or pulse [27].

### Patient and public involvement

The plans for OxCaMS were presented at a public open day of the Oxford cognitive health Clinical Research Facility and NIHR Oxford Health Biomedical Research Centre. Patients were not involved in the design of the study.

### Participants

Participants aged 18–35 years are recruited via advertisement or from the Oxford Student Sleep Survey [28]. Inclusion criteria include scoring ≥ 7 on the Mood Disorder Questionnaire (MDQ) [29, 30], indicating a history of periods of unstable and elevated mood and reporting that these episodes have caused at least mild functional impairment. In a separate study, we have shown that an MDQ score of > 7 correlates strongly with current, day-to-day mood variability (LZA, ACN, CHH, PJH, unpublished observations).

Participants meeting the MDQ criterion are initially screened by online questionnaire to assess for exclusion criteria including use of psychotropic medication currently or within the past 12 weeks; any contraindication to nicardipine, or to MRI or MEG; and reported harmful use of alcohol or illicit drugs.

### Baseline screening

Participants who pass the initial screening are invited to a pre-randomisation visit at which they are assessed for current or past psychiatric illness using the Mini International Neuropsychiatric Interview, version 5.0. Those meeting criteria for a disorder are not excluded, unless the clinician feels it might compromise safety or data quality.

Demographic details are recorded and a number of screening tools completed, including the McLean Screening Instrument for Borderline Personality Disorder [31] and the Adult Attention Deficit Hyperactivity Disorder (ADHD) Self-Report Scale (ASRS-v1.1). Alcohol consumption is assessed using the Alcohol Use Disorders Identification Test (AUDIT). Several self-report questionnaires are completed via an iPad, including the Barratt Impulsiveness Scale, the Affective Lability Scale (short form), the Affect Intensity Measure, the Morningness-Eveningness Questionnaire [32] and the Sleep Condition Indicator (SCI-R; [33]). A physical examination is carried out and vital signs and an electrocardiogram recorded. Blood is taken for routine analyses (including full blood count, urea and electrolytes, thyroid function, inflammatory markers) and for calcium channel gene expression and calcium signalling studies. Women of childbearing age have a pregnancy test. Participants also complete a battery of cognitive tasks and are instructed in the use of an iPad for completion of daily cognitive testing and mood monitoring, as described in the following sections. With the participants’ consent, their general practitioners are informed of their involvement in the study.

### Cognitive testing

A range of novel and standardised tasks measure aspects of cognition twice during the study—at baseline screening and at study endpoint:

- The theory of visual attention (TVA) framework quantifies fundamental parameters of information processing and attention: visual perception threshold, processing speed, capacity of visual short-term memory, attentional control and spatial bias of attention [34]. The TVA framework has been used widely in healthy participants [35–37] and in various cognitively impaired clinical populations [38, 39], but not in mood disorders.
- Two tasks from the emotional test battery (ETB) [40] examine emotional processing: the facial expression recognition test and emotional word categorisation. These tasks have been used in multiple experimental medicine studies and have been found to be reliable predictors and markers of antidepressant treatment response.
- The Stop-Signal Task assesses impulsivity and behavioural inhibition [41]. Stop-signal reaction times have been shown to increase with impulsivity and to vary across mood episodes of bipolar disorder [42].
- The N-Back task of working memory. This task is well validated, and performance is sensitive to drug manipulation (see e.g. [43]).
- The National Adult Reading Test (NART; [44]) is used as a measure of verbal IQ.

In addition to these tests, each day during the 4-week study period participants complete three novel app-based games on an iPad which they are given, delivered via the True Colours platform:

- The first is a simple gambling game (‘Wheel of Fortune’). On each trial, participants choose between two wheels, each divided into two segments proportional to the probability of winning or losing the amount of money specified in the segment. The aim of the task is to maximise the amount of ‘money’ that can be won over a session (total of 20 trials).
- The second game, ‘Fractals’, assesses reward learning. Each fractal is associated with a constant reward magnitude and reward probability. Participants learn to pick the most rewarding stimulus, ideally by integrating information about these two parameters. Each day participants are presented with 10 pairwise choices and 5 free choices in which all fractals are available to choose. In addition, participants are asked to guess how many points each fractal is worth (‘reward magnitude’) and how likely it is that points will be won (‘reward probability’ scale). Participants are allocated one set of 5 fractals before randomisation, and a new set of fractals after randomisation, to assess any differences in learning associated with nicardipine administration.
- The final game, ‘Whack-A-T’, assesses contextual cueing and implicit learning. It is based on a previously published task [45]. Participants are presented with an array of 11 ‘L’s and one ‘T’ and asked to tap on the ‘T’ as quickly as possible. There are three conditions: (1) novel — in which 20 different configurations are presented per day but never repeated, (2) daily — in which 20 different configurations are presented once per day, every day and (3) within-day — in which there are 20 repetitions of a configuration within a day, but they are not repeated on another day.

Happiness is rated before and after each game on a 21-point scale, from unhappy (− 10), through neutral (0), to happy (10). Participants are emailed each day with a reminder to complete the tasks. Adherence is monitored via the True Colours system (see below); if a participant misses tasks for 2 or more days in succession, he/she is prompted by the study team.

### Mood instability

Two measures of mood are recorded throughout the study. The Positive and Negative Affect Schedule, short form (PANAS-SF) is used for twice daily mood ratings. This 10-item self-report scale, which participants complete on the iPad, has high validity and reliability [46, 47] and has been widely used in healthy volunteer studies. To complement the PANAS ratings, the True Colours online self-management system (https://oxfordhealth.truecolours.nhs.uk/www/en/) is used to collect weekly mood ratings using clinically validated scales: the Quick Inventory of Depressive Symptomatology, self-report (QIDS-SR_16_) [48], the Altman Self-Rating Mania Scale [49], the Generalised Anxiety Disorder assessment (GAD-7) [50] and the EuroQol 5-dimension (EQ-5D) quality of life measure [51]. Our primary interest is in the effect of calcium channel antagonism on mood instability rather than in mood per se (although the latter is also recorded); instability will be quantified using statistical approaches described later.

### Sleep

Sleep is measured in a number of ways. As noted, participants complete the SCI-R and the Morningness-Eveningness Questionnaire at study baseline and at the final visit. They are also provided with a sleep diary for the study duration. These self-report sleep data will supplement the sleep data collected from ambulatory physiological monitoring (see subsequent sections).

### Actigraphy

Wrist-worn GENEActiv accelerometers are worn by participants for the study duration to continuously measure activity, light and body temperature. The data will help us to estimate the duration, timing and quality of sleep, as well as provide information about physical activity and light exposure.

### Remote ECG and blood pressure monitoring

Remote ECG is captured using the ePatch, a small, body-worn sensor that adheres to the skin. An ePatch will be fitted to participants at the baseline screening visit to wear for up to 72 h (maximum recording time). A second ePatch is provided to participants at the randomisation visit, with instruction to apply it 3 days after commencing nicardipine or placebo. The ePatch data allow us to explore the effect of nicardipine on heart rate variability and how this relates to mood instability and activity [52]. Participants are also given a portable blood pressure monitor and asked to record their daily blood pressure to assess any effects of nicardipine over the course of the randomised phase.

### LTCC gene expression and calcium signalling

Circulating leukocytes variably express calcium channel genes [53], and calcium signalling plays a central role in leukocyte activation and cytokine release. LTCCs in particular modulate activation of specific T cell subsets [54]; thus, nicardipine may have direct effects on T cell function and cytokine production. Moreover, peripheral inflammation has been implicated in mood disorders [55].

We will assess the effects of nicardipine on leukocyte function. First, LTCC expression in blood (in the samples taken at baseline and during the randomised phase) will be measured using quantitative reverse transcriptase-polymerase chain reaction for the encoding messenger RNAs. We will also assess the suitability of available LTCC subunit antibodies for immunoblotting. Second, we will determine the effect of nicardipine on intracellular free calcium stores and calcium transients in peripheral blood mononuclear cells. Basal calcium levels and stimulated calcium transients will be measured separately in monocytes and T cells, identified by immunolabelling, using a fluorescence-activated cell sorting method that utilises Fura Red calcium dye [56]. We will also assay circulating levels of interleukin-6 (IL-6) and other inflammatory biomarkers.

### Functional magnetic resonance imaging

Functional magnetic resonance imaging (fMRI) is used to explore the effects of calcium channel antagonism on neural dynamics and their variability, assessed using the blood oxygenation level dependent (BOLD) response. Participants are scanned at rest (eyes open) and whilst performing the gambling task described previously (~ 30 min) and a gender discrimination emotional facial expression task (~ 12 min). We include an arterial spin labelling (ASL) sequence to help distinguish cerebrovascular from neural effects of nicardipine.

MRI is carried out on a Siemens 3 T Trio with a 32-channel head coil. Visual stimuli are presented on a liquid-crystal display (LCD) screen (BOLDScreen 32; Cambridge Research Systems, Rochester, UK) visible to the participants through a mirror attached to the head coil. Physiological measures such as pulse and respiration are collected using BIOPAC. Each participant has two MRI scans, the first in week 1 (i.e. before randomisation) and the second during week 4.

Analyses will implement methods from the Centre for Functional MRI of the Brain (FMRIB) Statistical Library (FSL) [57]. Resting state connectivity will be investigated using independent component analysis (ICA) with the Multivariate Exploratory Linear Decomposition into Independent Components (MELODIC) tool. Evaluation of the resulting independent components will be used to compare neural networks of interest in the drug and placebo groups. We will apply a number of other FSL tools, including the fMRI Expert Analysis Tool (FEAT), an interface for model-based analysis of task fMRI based on general linear modelling (GLM). We will also utilise novel approaches being established by colleagues at the Wellcome Centre for Integrative Neuroimaging.

### Magnetoencephalography

Magnetoencephalography (MEG) is included in OxCaMS for three reasons. First, its temporal resolution and ability to detect rapid neural oscillations and transient networks [58] make it well suited to investigate whether these domains are affected by LTCC antagonism. Second, the MEG signal derives directly from neuronal activity, free of the potential vascular confounds of the BOLD signal in fMRI. Third, the MEG signal arises primarily from the synchronised excitatory post-synaptic potentials generated in cortical pyramidal neurons, and the LTCCs targeted by nicardipine are primarily expressed on neuronal dendrites and influence their depolarisation. Hence, a change in MEG signal can be interpreted as indicative of significant functional occupancy of neuronal LTCCs by nicardipine, serving as a marker of target engagement.

MEG data are acquired on a passively shielded Elekta Neuromag system with participants in the seated position. Visual stimuli are presented via a PROPixx projection system (VPixx). Visual fixation and blinks are monitored throughout the scan using an infrared eye tracker (Eyelink 1000). Electrocardiograms and electro-oculograms are collected to enable removal of physiological artefacts from the data during analysis. The MEG protocol begins with a 10-min, eyes open and fixated, resting state scan. Immediately following this, participants complete a precision working memory task combined with a retrocue manipulation, adapted from Mok et al. [59] (~ 35 min). They will also undergo a passive viewing task to induce visual gamma using a paradigm adapted from a previously published task [60] (~ 15 min) and complete a gripper task to measure cortico-muscular coherence (~ 15 min). As with MRI, each participant has two MEG scans, the first in week 1 (i.e. before randomisation) and the second in week 4.

Analysis of resting state MEG measurements will explore the effect of LTCC antagonism on oscillatory activity and the integrity of resting state functional networks in mood instability. Data will be interrogated in both sensor space and source space after applying a linearly constrained minimum variance beamformer. Metrics such as oscillatory power and peak frequency will be explored. Additionally, we will apply hidden Markov modelling to identify transient brain states and assess any effects of nicardipine upon the dynamics of states (e.g. frequency of state switching and their fractional occupancy). Differences in drug/placebo groups will be tested using GLM. We will also utilise novel analytical approaches to MEG being established by colleagues at the Wellcome Centre for Integrative Neuroimaging (see e.g. [58, 61]).

### Final visit

After 14 days of randomisation to nicardipine or placebo, participants attend a final visit. A number of procedures/measurements are repeated from the screening visit, including physical examination and vital signs, blood samples, ECG and re-administration of the questionnaires and cognitive testing (with the exception of the NART). The GENEActiv, blood pressure, ePatch and iPad devices will be collected. Return of nicardipine and placebo is carried out by Clinical Research Facility staff. Participants fill out a number of questionnaires assessing the tolerability and acceptability of all devices and questions assessing interpretation of the mood questionnaires and cognitive tasks. To test the success of blinding, participants are asked to guess their group allocation. The second MRI and MEG scans, as detailed previously, are repeated either at the final visit or in the week before this visit, dependent upon scanner and participant availability.

### Power and statistical approaches

OxCaMS is an exploratory experimental medicine study, and we will investigate the effect of LTCC antagonism on a range of parameters. As the first study of its kind, no meaningful power calculations are possible. The planned sample size (*n* = 20 in each group) is intended to be feasible and is similar to those of many comparable experimental medicine studies in psychiatry, including one in which the effects of lithium carbonate on cognitive and neural markers in healthy volunteers were examined. This suggested a sample size of 40 with 80% power at alpha = 0.05 (see also [62]). OxCaMS is larger than the other experimental studies which have used an LTCC antagonist to examine cerebral blood flow (*n* = 12 [63], *n* = 8 [64]) and MRI indices of brain hypoxia (*n* = 17 [65]). OxCaMS is also of similar or greater size than the studies which have demonstrated the effects of nicardipine or other LTCC antagonists on gastrointestinal motility (*n* = 42 [28], *n* = 12 [66]) and pressor responses to exercise (*n* = 12 [67]).

The outcomes to be investigated are summarised in Table 2. The specified primary outcome is on cognitive stability, across the range of tasks described above. Key secondary outcomes comprise the effect of LTCC antagonism on mood and neural markers and their stability, on sleep and on the peripheral markers of calcium channels. In addition, participants will be genotyped for rs1006737, the index risk single nucleotide polymorphism within *CACNA1C*, using a TaqMan assay. Risk and non-risk allele carriers differ in Ca_V_1.2 expression and activity [68], and this may influence their response to LTCC antagonism [69]. Hence, although our power will be very limited, we will examine for effects of genotype in exploratory analyses. Participation in the study is not based on genotype, since the low frequency of the minor allele (~ 0.30) would make recruitment based on homozygosity unfeasible, given time and resources available.

**Table 2:** Summary of outcome measures for OxCaMS

*Primary outcome*	
The effect of L-type voltage-gated calcium channel (LTCC) antagonism on cognitive instability	
*Secondary outcomes*	
The effect of LTCC antagonism on mood instability	
The effect of LTCC antagonism on physical activity and sleep	
The effect of LTCC antagonism on neural dynamics, at rest and during task performance	
The effect of LTCC antagonism on heart rate variability	
The effect of LTCC antagonism on calcium channel expression and calcium signalling	
The effect of rs1006737 genotype and its interaction with LTCC antagonism on the above measures	

Data will be inspected for normality. For data meeting this criterion, group (drug vs. placebo) comparisons for longitudinal measures will utilise repeated measures analysis of variance (ANOVA); simple between-group comparisons will be made using unpaired *t* tests; correlations between variables will be explored using Pearson correlations. Data which are non-Gaussian will be explored for outliers, and either transformed or subjected to non-parametric analysis.

Our primary measure of instability for cognitive and mood indices will be the root mean square of successive differences, a widely used measure of instability (see e.g. [70, 71]). We will also explore additional mathematical approaches for the evaluation of instability (e.g. entropy, time signatures). MRI and MEG analyses have been outlined above.

### Other issues

Like other LTCC antagonists, nicardipine can produce a range of side effects, of which headache, flushing and ankle oedema are prominent. Side effects are reported in 11–14% of patients [72], but they are generally minor and transient and are less common with the SR formulation (Cardene SPC, Roche). Nevertheless, their occurrence may compromise the blindness of treatment allocation and may cause drop-outs.

We anticipate that some participants will meet diagnostic criteria for a psychiatric disorder, especially bipolar disorder. If so, the participant will be told and will receive information and advice. With the participant’s consent, their general practitioner will also be informed. The criterion of exclusion due to harmful use of alcohol was introduced to help exclude subjects in whom mood instability is driven primarily by their use of alcohol. We realise that this is an imperfect criterion, and it means that our results may not generalise to the many people who have mood instability and who do have harmful use of alcohol and other substances.

We intend to publish all results of OxCaMS in open access journals. Brief summaries will be posted on our websites.

## Discussion

OxCaMS will help establish whether a 14-day run of LTCC blockade produces effects on brain and behaviour when used in adults with mood instability and, if so, the nature of these effects. It is an exploratory study which examines a range of parameters and tests a number of hypotheses related to the proposed role of LTCCs in relevant domains of brain function. By its nature, it is not expected that definitive or clinically actionable results will arise; rather, OxCaMS is designed to provide findings which can help inform future studies of LTCC antagonists for psychiatric indications, as well as to show the feasibility of a high-intensity, multimodal experimental medicine study.

Demonstration of beneficial effects of nicardipine on any of the indices will encourage consideration of clinical trials to test LTCC antagonists in specific psychiatric patient groups. However, even if this is not observed, bear in mind that the current class of LTCC antagonists were developed specifically for their cardiovascular effects, and that much of the underlying pharmacology was carried out in rodents. It is increasingly clear that there are significant differences in the composition of LTCCs between brain and periphery and between rodents and humans; hence, it may be possible to develop modified drugs of this class which have a greater selectivity for, and efficacy at, neuronal LTCCs. In this context, OxCaMS will serve as a proof of principle as to whether a psychiatric ‘signal’ can be observed with existing LTCC antagonists, albeit one which is likely to be therapeutically suboptimal. More broadly, OxCaMS will provide an exemplar of how experimental medicine studies are one way in which the treatment potential of psychiatric genomic discoveries, in this instance regarding LTCC genes, can be investigated.

## Trial status

The trial protocol is version 1.5, dated 23 May 2018.

Recruitment began on 19 December 2017; anticipated completion of recruitment is December 2019.

## Additional files

Additional file 1: SPIRIT checklist. (DOCX 52 kb)

Additional file 2: Study protocol, current version. (DOCX 1508 kb)

Additional file 3: Ethical approval letter. (DOCX 846 kb)

## Abbreviations

ASRM: Altman Self-Rating Mania Scale
ASL: Arterial spin labelling
AUDIT: Alcohol Use Disorders Identification Test
ETB: Emotional test battery
fMRI: Functional magnetic resonance imaging
LTCC: L-type calcium channel
MDQ: Mood Disorder Questionnaire
MEG: Magnetoencephalography
NART: National Adult Reading Test
PANAS: Positive and Negative Affect Schedule
QIDS-SR: Quick Inventory of Depressive Symptomatology (self-report)
SCI: Sleep Condition Indicator
