# Supplementary material for: The Oxford study of Calcium channel Antagonism, Cognition, Mood instability and Sleep (OxCaMS): study protocol for a randomised controlled, experimental medicine study
Source: Trials. 2019 Feb 12;20:120. doi: 10.1186/s13063-019-3175-0 (PMC6373140; doi:10.1186/s13063-019-3175-0)
Supplement: Supplementary file 2 — Study protocol, current version. (DOCX 1508 kb) [file 13063_2019_3175_MOESM2_ESM.docx]

**Study title: OxCaMS: The Oxford Study of Calcium Channel Antagonism, Cognition, Mood Instability and Sleep.**

**Short title: OxCaMS**

| **NHS REC Ref:**  **IRAS Ref.**  **Date and Version:** | 17/SC/002  213212  V 1.5 (23/05/2018) | |  |
| --- | --- | --- | --- |
| **Chief Investigator:** | | Professor Paul Harrison,  University of Oxford Department of Psychiatry,  Warneford Hospital, Oxford, OX3 7JX  Tel: 01865 618329, Fax: 01865 251076.  Email paul.harrison@psych.ox.ac.uk | |
| **Investigators:** | | Professor John Geddes*  Professor Catherine Harmer* Professor Kia Nobre*  Dr Kate Saunders* Dr Jennifer Rendell* Professor Andrea Cipriani*  Dr Mary Jane Attenburrow* Helen Jones^##^  Lauren Atkinson* Lucy Colbourne*  Dr Elizabeth Tunbridge*  Dr Riccardo Giorgio  Dr Alexander Smith  Arne Mould*  Simon Bond*  * University of Oxford Department of Psychiatry ^##^  Oxford Health NHS Foundation Trust | |
| **Sponsor:** | | University of Oxford | |
| **Funder:** | | Wellcome Trust strategic awards, reference: 102616/Z/13/Z and 098461/Z, NIHR Oxford Health Biomedical Research Centre | |
| **Chief Investigator:** | | Signature: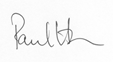*.* Date: 23/05/2018 | |

**Potential conflicts of interest*: None***

**Confidentiality Statement**

This document contains confidential information that must not be disclosed to anyone other than the authorised individuals from the University of Oxford, the Investigator Team and members of the NHS Research Ethics Committee), unless authorised to do so.

**CONTENTS**

[1. KEY STUDY CONTACTS 6](#_Toc467666493)

[2. SYNOPSIS 7](#_Toc467666494)

[3. ABBREVIATIONS 10](#_Toc467666495)

[4. BACKGROUND AND RATIONALE 11](#_Toc467666496)

[4.1. Calcium & bipolar disorder 11](#_Toc467666497)

[4.2. Study themes 12](#_Toc467666498)

[4.3. Summary 13](#_Toc467666499)

[5. OBJECTIVES AND OUTCOME MEASURES/ENDPOINTS 13](#_Toc467666500)

[6. STUDY DESIGN 14](#_Toc467666501)

[6.1. Visit 1- Pre-randomisation phase 14](#_Toc467666502)

[6.2. Visit 2 - Randomisation visit 14](#_Toc467666503)

[6.3. Randomised phase visits 14](#_Toc467666504)

[6.4. Visit 3 – Final visit: 15](#_Toc467666505)

[6.5. Post-study follow-up 15](#_Toc467666506)

[7. PARTICIPANT IDENTIFICATION 15](#_Toc467666507)

[7.1. Study Participants 15](#_Toc467666508)

[7.2. Inclusion Criteria 15](#_Toc467666509)

[7.3. Exclusion Criteria* 15](#_Toc467666510)

[8. STUDY PROCEDURES 16](#_Toc467666511)

[8.1. Recruitment 16](#_Toc467666512)

[8.2. Informed Consent 16](#_Toc467666513)

[8.3. Visit 1 - Pre-randomisation phase 17](#_Toc467666514)

[8.4. Visit 2 - Randomisation 18](#_Toc467666515)

[8.5. Visit 3 – final visit 18](#_Toc467666516)

[8.6. Post-study follow-up 19](#_Toc467666517)

[8.7. Randomisation and allocation concealment 19](#_Toc467666518)

[8.8. Minimisation variables 19](#_Toc467666519)

[8.9. Unblinding of treatment 19](#_Toc467666520)

[8.10. Sample Handling 20](#_Toc467666521)

[8.11. Discontinuation/Withdrawal of Participants from Study Medication 20](#_Toc467666522)

[8.12. Definition of End of Study 20](#_Toc467666523)

[8.13. Description of procedure 21](#_Toc467666524)

[9. NICARDIPINE 21](#_Toc467666525)

[9.1 Description 21](#_Toc467666526)

[9.2 Storage 21](#_Toc467666527)

[9.3 Compliance with Study Medication 22](#_Toc467666528)

[9.4 Accountability of the Study Medication 22](#_Toc467666529)

[9.5 Concomitant Medication 22](#_Toc467666530)

[10. SAFETY REPORTING 23](#_Toc467666531)

[10.1. Definitions 23](#_Toc467666532)

[10.2. Procedures for Recording Adverse Events 24](#_Toc467666533)

[10.3. Reporting Procedures for Serious Adverse Events 24](#_Toc467666534)

[10.4. Expectedness 24](#_Toc467666535)

[10.5. SUSAR Reporting 24](#_Toc467666536)

[10.6. Steering Committee 25](#_Toc467666537)

[11. ANALYSES 25](#_Toc467666538)

[11.1. Description of Statistical Methods 25](#_Toc467666539)

[11.2. Criteria for the Termination of the Study 26](#_Toc467666540)

[11.3. Procedure for Accounting for Missing, Unused, and Spurious Data. 26](#_Toc467666541)

[11.4. Inclusion in Analysis 26](#_Toc467666542)

[12. DATA MANAGEMENT 26](#_Toc467666543)

[12.1. Source Data 26](#_Toc467666544)

[12.2. Access to Data 26](#_Toc467666545)

[12.3. Data Recording and Record Keeping 26](#_Toc467666546)

[13. QUALITY ASSURANCE PROCEDURES 26](#_Toc467666547)

[14. SERIOUS BREACHES 27](#_Toc467666548)

[15. ETHICAL AND REGULATORY CONSIDERATIONS 27](#_Toc467666549)

[15.1. Declaration of Helsinki 27](#_Toc467666550)

[15.2. Guidelines for Good Clinical Practice (GCP) 27](#_Toc467666551)

[15.3. Approvals 27](#_Toc467666552)

[15.4. Reporting 27](#_Toc467666553)

[15.5. Participant Confidentiality 27](#_Toc467666554)

[15.6. Expenses and Benefits 27](#_Toc467666555)

[15.7. Other Ethical Considerations 27](#_Toc467666556)

[16. FINANCE AND INSURANCE 30](#_Toc467666557)

[16.1. Funding 30](#_Toc467666558)

[16.2. Insurance 30](#_Toc467666559)

[17. PUBLICATION POLICY 30](#_Toc467666560)

[18. REFERENCES 31](#_Toc467666561)

[19. Appendix A: Participant pathway 34](#_Toc467666562)

[20. Appendix B: Schedule of Procedures 35](#_Toc467666563)

[21. Appendix C: SAE Reporting Flow Chart 38](#_Toc467666564)

[22. Appendix D: Rating scales 39](#_Toc467666565)

[23. Appendix E: Theme 2 Cognitive tests and neural dynamics 40](#_Toc467666566)

[24. Appendix F: Theme 3 Sleep, motor activity and social interactions 43](#_Toc467666567)

[25. Appendix G: Theme 4 Profile of the calcium signalling system 43](#_Toc467666568)

[26. Appendix I: ePatches 44](#_Toc467666569)

[27. Appendix J: Screening questionnaires 45](#_Toc467666570)

[Barratt Impulsiveness Scale (BIS-11) 45](#_Toc467666571)

[Sleep Condition Indicator (SCI-R) 46](#_Toc467666572)

[Maclean Screening Instrument (MSI-BPD) 47](#_Toc467666573)

[Affective Lability Scale – Short Form (ALS-SF) 48](#_Toc467666574)

[Affect Intensity Measure (AIM) 49](#_Toc467666575)

[28. Appendix K: Collaborator agreement 51](#_Toc467666576)

[29. Appendix M: Devices agreement 52](#_Toc467666578)

[30. Appendix L: Amendment History 52](#_Toc467666579)

# KEY STUDY CONTACTS

| **Chief Investigator** | Professor Paul Harrison Head of Department,  University of Oxford Department of Psychiatry  Warneford Hospital, Oxford, OX3 7JX Tel: 01865 618329 Fax: 01865 251076  Email: paul.harrison@psych.ox.ac.uk |
| --- | --- |
| **Sponsor** | Heather House  Head of Clinical Trials and Research Governance, Research Services, University of Oxford, Joint Research Office, Block 60, Churchill Hospital, Oxford, OX3 7LE Tel: 01865 572224  Email: [ctrg@admin.ox.ac.uk](mailto:heather.house@admin.ox.ac.uk) |
| **NIHR Oxford cognitive health Clinical Research Facility** | Helen Jones  NIHR CRF  Warneford Hospital  Oxford, OX3 7JX  Tel: 01865 902135 |
| **Steering Committee Chair** | Professor Ian Jones  Institute of Psychological Medicine and Clinical Neurosciences  Cardiff University School of Medicine  Hadyn Ellis Building  Maindy Road  Cathays  Cardiff CF24 4HQ    Tel: 0044 029 20 688 327  Email: JonesIR1@cf.ac.uk |

# SYNOPSIS

| **Aim** | To characterise the cognitive, neural and pathophysiological effects of calcium channel antagonism in healthy volunteers with mood instability. |
| --- | --- |
| **Study Title** | OxCaMS: The Oxford Study of Calcium Channel Antagonism, Cognition, Mood Instability and Sleep. |
| **Short Title** | OxCaMS |
| **Study Design** | Experimental medicine study using a randomised, double-blind, placebo-controlled design |
| **Study duration** | 01/10/2017 – 01/10/2019 |
| **Study Participants** | Healthy volunteers with Score of ≥7 on the MDQ with evidence of associated functional impairment  INCLUSION CRITERIA:   - Willing and able to given informed consent to participate in the study - Male or female - Aged 18 – 35 - Significant mood instability (defined as a score of ≥7 as measured by the Mood Disorder Questionnaire (MDQ)) - No indication that urgent psychiatric treatment is required - Pre-treatment tests including renal, cardiac and liver function acceptable for the initiation of treatment with nicardipine - Willing and able to comply with the study requirements - Willing to allow his/ her General Practitioner, if appropriate, to be notified of his/her participation in the study.   EXCLUSION CRITERIA:   - Contraindication(s) to nicardipine (as documented in the Summary of Product Characteristics for Cardene) - History or current axis 1 disorder, if in the opinion of the investigator it will compromise safety or affect data quality. - Regular psychotropic drug use within the last 12 weeks. Recent ‘as required’ use of psychotropic medication may be permitted at the investigators discretion, if it will not compromise safety or affect data quality. - Currently taking any other medication or herbal extracts that would affect study results or safety (e.g. St. John’s Wort). - Judged to be at significant immediate risk of suicide/self-harm - Clinically significant alcohol or substance use, if in the opinion of the investigator it will compromise safety or affect data quality - Requiring urgent treatment for an acute mood episode such that placebo would be inappropriate - Female and pregnant, lactating or planning a pregnancy during the course of the study - Female of child-bearing potential not willing to use effective contraception - Participation in a research study involving an investigational medicinal product in the past 12 weeks - Individuals who are lactose intolerant (due to Nicardipine containing lactose).   Plus: Participants who have a pacemaker, non-MR-compatible metal implant, or any other contraindication for MR or MEG brain scanning will be excluded from the corresponding brain scanning element(s) of the study  Individuals who are not willing to consume gelatine (due to drug and placebo capsules being made of gelatine).  ****** No participants will be withdrawn from effective medication or treatment for the purposes of this study |
| **Target sample size** | 40 randomised participants |
| **Treatment duration** | 2-week randomised phase |
| **Follow up duration** | 1 week |
| **Planned Accrual Period** | 2 years |
| **Intervention** | Nicardipine SR |
| **Formulation, Dose, Route of Administration** | Nicardipine sustained release 30mg taken twice daily.  Matched placebo. |

| **Objectives** | **Outcome Measures/Endpoints** |
| --- | --- |
| Primary objective:  To compare the effects of nicardipine and placebo on measures of cognitive instability.  . | Primary outcome measure: Cross sectional and longitudinal assessments of cognition |
| Secondary objectives  1. To compare the effects of nicardipine and placebo on variability in neural dynamics during MRI and MEG scans. This will include scans during resting state and whilst performing neuropsychological tasks.  2. To explore the effects of nicardipine on physical activity and sleep.  3. To develop a profile of calcium channel expression and calcium signalling with and without nicardipine treatment.  4.Heart rate variability  5. Mood instability | Secondary outcome measure   1. Blood oxygen level dependent signal during rest and during cognitive testing; induced and evoked field activity 2. Activity monitor measurements:   2a. Actigraphy data from a maximum of two wearable units, allowing analysis of indices of:   1. Frequency and amplitude of movements during daytime activity   ii. Categorisation of activity types.   1. Duration, timing, and quality of sleep.   2b. Sleep quality indicator questionnaire (SCI; self-report sleep measure (Espie, 2014)).  3. Measure effects of nicardipine on leucocyte calcium channel expression and calcium signalling  4. Change in R-R interval variability   1. Changes in mood instability as defined using root mean square of the successive differences (RMSSD) |

# ABBREVIATIONS

| ADHD | Attention deficit hyperactivity disorder |
| --- | --- |
| AE | Adverse event |
| ALTMAN | Altman Self Rating Scale for Mania |
| AR | Adverse reaction |
| AUDIT | The Alcohol Used Disorders Identification Test |
| BD | Bipolar Disorder |
| CI | Chief Investigator |
| CTRG | Clinical Trials and Research Governance |
| DSUR | Development Safety Update Report |
| ECG | Electrocardiogram |
| FSL | fMRIB Statistical Library |
| GCP | Good Clinical Practice |
| IB | Investigators Brochure |
| ICF | Informed Consent Form |
| ICH | International Conference of Harmonisation |
| IMP | Investigational Medicinal Product |
| LTCC | L-type calcium channel |
| LVLP | Last Visit Last Patient |
| MDQ | Mood Disorders Questionnaire |
| MEG | Magnetoencephalography |
| MINI | Mini-International Neuropsychiatric Interview |
| MRI | Magnetic Resonance Imaging |
| NGAL | Neutrophil Gelatinase-associated Lipocalin |
| NHS | National Health Service |
| NIHR | National Institute for Health Research |
| NIHR-CRF | NIHR cognitive health Clinical Research Facility |
| OHBA | Oxford **c**entre for Human Brain Activity |
| PANAS | Positive and Negative Affect Scale |
| PI | Principal Investigator |
| PIS | Participant Information Sheet |
| PRN | Pro re nata – refers to medication taken as required |
| QIDS-SR_16_ | Quick Inventory of Depressive Symptomatology |
| R&D | NHS Trust R&D Department |
| REC | Research Ethics Committee |
| SAE | Serious Adverse Event |
| SAR | Serious Adverse Reaction |
| SCI | Sleep Condition Indicator |
| SCID-I | The Structured Clinical Interview for Axis I Disorders |
| SCRD | Sleep and Circadian Rhythm Disruption |
| SCNI | Sleep and Circadian Neuroscience Institute |
| SDV | Source Data Verification |
| SmPC | Summary of Product Characteristics |
| SOP | Standard Operating Procedure |
| SUSAR | Suspected Unexpected Serious Adverse Reactions |
| TC | True Colours |
| TMF | Study Master File |
| Study-CRF | Study Case Report Form |
| TSC | Study Steering Committee |

# BACKGROUND AND RATIONALE

## Calcium & bipolar disorder

Calcium signalling is likely to play in important role in the pathophysiology of a number of psychiatric disorders, particularly bipolar disorder. Altered levels of calcium have been reported in cerebrospinal fluid in patients with mania, (Jimerson 1979), and long-term lithium treatment is associated with altered calcium metabolism, including hyperparathyroidism (Mcknight 2012). These findings have been complemented by the demonstration of altered calcium signalling in stem cells and neurons derived from patients with bipolar disorder compared to healthy controls, and in those who respond to lithium treatment compared to non-responders (Hahn 2005, Chen 2014, Mertens 2015, McCarthy 2016). These findings are complemented by the discovery of genome-wide significant association of BD with several calcium channel genes, notably the L-type calcium channel (LTCC) CACNA1C, which encodes Cav1.2 (Ferreira 2008, Craddock 2013). Rare variants are linked with bipolar disorder in multiply affected families. CACNA1C is further implicated by whole-genome sequencing of BD patients and controls (Fiorentino 2014), and by its altered expression in the frontal cortex of patients with bipolar disorder. Separate work strongly implicates CACNA1C in memory and neural plasticity (Moosmang 2005). CACNA1C and other LTCC subunits form part of the genetic contribution to cognition (Heck 2015) and sleep (Byrne 2013, Parsons 2013). Both cognitive dysfunction and sleep disturbance are widely reported in BD and persist in euthymia (Bourne 2013, Harvey 2005). Sleep deprivation is a common precipitant for mania and hypersomnia is present in up to 80% of those with bipolar depression (Harvey 2008). The CACNA1C genotype may also influence other domains relevant to bipolar disorder and its therapy, including resilience, depressive symptoms, and reward responsiveness (Lancaster 2014, Strohmaier 2013). These landmark genetic findings have clear therapeutic relevance. Firstly, LTCC antagonists are licensed for hypertension and angina, and are thus available for immediate use in experimental and clinical studies. Secondly, lithium and lamotrigine, which have established long-term efficacy in BD, both modulate calcium signalling amongst their other actions. Thirdly, our recent systematic review shows that LTCC antagonists may have some efficacy in mania (Cipriani 2016) although there is a paucity of data from randomised controlled trials (RCT). Initial data (from brain, and from induced neurons) indicate that the risk variant in CACNA1C likely involves a gain of function (Yashimuzu 2015). This is consistent with the biochemical evidence in BD and suggests that antagonism is therapeutically desirable.

While these recent developments are promising there remains considerable uncertainty as to the neural or cognitive effects of LTCC antagonists such that a randomised controlled study in clinical populations cannot be justified. In order to address this absence of evidence we are proposing an experimental medicine study using a randomised placebo controlled design. Our goal is not intended to investigate efficacy per se but to use LTCC antagonism as a vehicle to explore the role of calcium channels in cognitive and neural variability in a population of healthy volunteers with mood instability as defined by the mood disorders questionnaire (MDQ). Mood instability is associated with sleep problems ( McDonald 2017), impaired cognition (Bourne 2013) and altered neural connectivity (Broome 2015), so it seems likely that this group will be more sensitive to the effect of LTCC antagonism.

## Study themes

The main focus of OxCaMS is the effect of nicardipine on variability in cognitive function as measured using daily cognitive tasks.

We will also explore the impact of nicardipine on, behaviour (including activity levels and sleep patterns), neural activity and circadian rhythms. Exploration of the effects of LTCC antagonists on these parameters and the correlation between these effects form a secondary focus for the study.

-**Regular cognitive tasks**. Brief cognitive tasks will be presented daily to participants on an iPad. These tasks will explore aspects of attention, reinforcement learning, working memory and risk sensitivity and will take around 10 minutes to complete each day. These paradigms are already being used in a number of other studies in healthy volunteers with mood instability (http://conbrio.psych.ox.ac.uk/comet).

-**Actigraphy**. Participants will be given a wrist mounted actigraph to wear for the duration of the study. In addition to movement the actigraph also collects data on ambient temperature and light. This will allow us to monitor movement and sleep with a high degree of accuracy.

-**Functional magnetic resonance imaging (fMRI).** Resting state and task-related functional imaging will be performed. Arterial spin labelling (ASL) sequences will also be performed. ASL is a non-invasive means of quantifying tissue perfusion (Petcharunpaisan 2010).

-**Magentoencephalography (MEG).** Resting state and task-related imaging will be performed.

- **Blood samples for genotyping** for the major BD-associated allele in *CACNA1C* (rs1006737). Blood samples will also be used to measure leucocyte calcium channel gene expression and studies of calcium signalling.

- **Heart rate variability** will be measured prior to and during the randomised phase using an e-Patch device [41]. This is an unobtrusive monitor, which provides a 72-hour heart rhythm trace. Heart rate variability is of interest because of the cardiovascular effects of nicardipine. (Bassett 2015).

Mood instability: this will be measured using root mean square of the successive differences (RMSSD). This is a standardised measure of instability that has been used in a number of studies

Participant will also be asked to complete daily and weekly measures of mood in order to allow us to control for any changes is mood as we are wanting to explore the direct effect of calcium channel antagonism unconfounded by mood. Daily mood will be recorded using the short form of the positive and negative symptom scale (PANAS [37]) delivered on an iPad. The PANAS 10 item is a self-report scale which is widely used in psychiatric research. Weekly mood measures will be completed using the True Colours system ([www.truecolours.nhs.uk](http://www.truecolours.nhs.uk)). True colours is a mood monitoring platform developed in Oxford which is uses validated clinical scales to assess depression (quick inventory of depressive symptoms) and mania (Altman mania rating scale [39]).

All participants will have a two-week period of participation prior to randomisation. This will allow us to assess the stability of their cognitive function and collect baseline mood, sleep and activity data.

## Summary

The use of the range of paradigms outlined above to explore early cognitive neural and behavioural, effects will greatly increase understanding of the mechanism of action of LTCC antagonists. This understanding will inform whether LTCC antagonists may be efficacious in bipolar disorder and have implications for future drug development.

# OBJECTIVES AND OUTCOME MEASURES/ENDPOINTS

| **Objectives** | **Outcome Measures/Endpoints** |
| --- | --- |
| Primary objective:   - To compare the effects of nicardipine and placebo on measures of cognitive instability.   . | Primary outcome measure:   - Cross sectional and longitudinal assessments of cognition |
| Secondary objective:   - To compare the effects of nicardipine and placebo on variability in neural dynamics during MRI and MEG scans. This will include scans during resting state and whilst performing neuropsychological tasks. - To explore the effects of nicardipine on physical activity and sleep. - To develop a profile of calcium channel expression and calcium signalling with and without nicardipine treatment - Heart rate variability - Mood instability | Secondary outcome measure:   - Blood oxygen level dependent signal during rest and during cognitive testing; induced and evoked field activity - Activity monitor measurements:   2a. Actigraphy data from a maximum of two wearable units, allowing analysis of indices of:   1. Frequency and amplitude of movements during daytime activity   ii. Categorisation of activity types.   1. Duration, timing, and quality of sleep.   2b. Sleep quality indicator questionnaire (SCI; self-report sleep measure (Espie, 2014)).   - Measure effects of nicardipine on leucocyte calcium channel expression and calcium signalling - Change in R-R interval variability - Change in mood instability using root mean square of the successive differences (RMSSD) |

# STUDY DESIGN

Experimental medicine study using a randomised, 2-week, double-blind, placebo-controlled design.

## Visit 1- Pre-randomisation phase

Participants who give Informed Consent and have current mood instability with evidence of associated functional impairment will enter a pre-randomisation phase. General health as well as any medication taken will be reviewed. In addition they will be assessed using the MINI International Neuropsychiatric Interview (MINI), complete a battery of cognitive tests, be provided with an iPad and activity monitor(s) and be set up to provide information about their mood, cognitions and activities throughout the study. Vital signs will be checked, blood samples, and an ECG performed. Female participants will also be asked to take a pregnancy test. Participants will be asked to wear an ePatch (an ambulatory ECG monitor) for 72-hours, complete a sleep questionnaire and keep a sleep diary.

The pre-randomisation phase will enable participants to become familiar with True Colours, PANAS and complete the cognitive tests prior to randomisation. The duration of this phase will be approximately 2 weeks, after which we will schedule pre-treatment MRI and MEG scans and the randomisation visit.

## Visit 2 - Randomisation visit

At the end of the pre-randomisation phase participants will attend a randomisation visit when they will be randomly allocated oral nicardipine or matched placebo capsules in the ratio of 1:1. The randomised phase will last for 2-weeks. At this visit participants will be asked to have a MRI and a MEG scan prior to commencing treatment. Both scans will include resting state and functional task related scans. In addition, participants will be provided with an ePatch for the second 72-hour measurement period and asked to complete the Sleep Questionnaire and sleep diary following this period. They will also be given a blood pressure monitor to monitor their blood pressure throughout the two week randomisation phase . Appointments will be made for the final visit and MRI and MEG scans during week 4.

## Randomised phase visits

During the randomised phase participants will continue with questionnaires and cognitive tests as during the pre-randomisation phase. They will not be asked to come in for any additional visits unless participants have concerns or questions relating to the study.

## Visit 3 – Final visit:

At approximately 2 weeks post-randomisation participants will be asked to attend a final study visit. At this visit, vital signs will be checked, and ECG and blood samples will be repeated. Participants will also complete a second battery of cognitive tasks. All equipment will be returned at this visit including medication bottles and any remaining medication. A post-treatment MRI scan and MEG scan will be done. If possible, post randomisation scans and the final week 2 visit will be carried out on the same day. If this is not possible due to scanner availability, separate visits will be scheduled at the convenience for the participant.

At this visit checks of adherence and for adverse events will be carried out.

## Post-study follow-up

Participants will be contacted following cessation of study treatment to review the status of any reported adverse events (AEs) and to check for any unreported AEs.

Please see Appendix A for a diagram of the participant pathway in the study and assessments at each visit.

# PARTICIPANT IDENTIFICATION

## Study Participants

Participants will be individuals with current mood instability who demonstrate evidence of associated functional impairment and have been recruited through student surveys or from advertisement in the community.

## Inclusion Criteria

- Willing and able to give informed consent to participate in the study
- Male or female
- Aged 18 - 35
- Clinical complaint of significant mood instability (≥7 as measured by the Mood Disorder Questionnaire (MDQ))
- No indication that urgent psychiatric treatment is required
- Pre-treatment tests including renal, cardiac, thyroid and parathyroid functions acceptable for initiation of treatment with nicardipine
- Willing and able to comply with all study requirements including mood and behavioural monitoring (True Colours) and MRI and MEG scanning and blood tests (assessed by a psychiatrist).
- Willing to allow his/ her General Practitioner and consultant, if appropriate, to be notified of his/her participation in the study.

## Exclusion Criteria*

- Contraindication(s) to nicardipine (as documented in the Summary of Product Characteristics for Cardene)
- History or current axis 1 disorder, if in the opinion of the investigator it will compromise safety or affect data quality.
- Regular psychotropic drug use within the last 12 weeks. Recent ‘as required’ use of psychotropic medication may be permitted at the investigators discretion, if it will not compromise safety or affect data quality.
- Currently taking any other medication or herbal extracts that would affect study results or safety (e.g. St. John’s Wort).
- Judged to be at significant immediate risk of suicide/self-harm
- Clinically significant alcohol or substance use, if in the opinion of the investigator it will compromise safety or affect data quality
- Requiring urgent treatment for an acute mood episode such that placebo would be inappropriate
- Female and pregnant, lactating or planning a pregnancy during the course of the study
- Female of child-bearing potential not willing to use effective contraception
- Participation in a research study involving an investigational medicinal product in the past 12 weeks
- Individuals who are lactose intolerant (due to nicardipine containing lactose).

Plus

- Participants who have a pacemaker, non-MR-compatible metal implant, or any other contraindication for MR or MEG brain scanning will be excluded from the corresponding brain scanning element(s) of the study.
- Individuals who are not willing to consume gelatine (due to drug and placebo capsules being made of gelatine).

***** Participants with a primary diagnosis of bipolar disorder with co-morbid anxiety or borderline

Personality disorder are not excluded.

****** No patient will be withdrawn from effective medication or treatment for the purposes of this
 study

# STUDY PROCEDURES

## Recruitment

The following recruitment strategies will be employed:

*Student survey:* The Department of Psychiatry regularly sends surveys to Oxford students which include the Mood Disorder Questionnaire (MDQ. Respondents to that survey whose MDQ scores indicate possible significant mood instability and who stated their willingness to consider participating in research will be sent the participant information sheet and invited to a research assessment interview.

*Publicity:* Posters and leaflets will be placed in the local community and in university departments in Oxford and information about the study will be made available online. Online adverts will be placed on websites such as daily info ([www.dailyinfo.org](http://www.dailyinfo.org)) and Gumtree. Adverts will also be hosted on TV screens, on local bus services and posters added on public notice boards. People who express an interest in the study will be emailed an electronic version of the patient information sheet and the MDQ to complete. Those whose scores indicate that they have experienced significant mood instability (≥7) will be invited to a research assessment interview as above. For participation in the study, all participants must fulfil all inclusion and exclusion criteria.

## Informed Consent

Participants that have expressed an interest in the study will be emailed a copy of the Participant Information Sheet (PIS). At visit 1, where appropriate, Informed Consent will be obtained.

Written and verbal versions of the PIS and Informed Consent Form will be presented to the patient detailing the exact nature of the study, what it would involve, the implications and constraints of the protocol, the known adverse effects and any risks involved in taking part. It will be clearly stated that the patient would be free to withdraw from the study at any time for any reason without prejudice to future care, and with no obligation to give the reason for withdrawal.

Participants will be allowed as much time as they wish to consider the information and be given the opportunity to question the study team their GP or other independent parties to decide whether they will participate in the study. Written Informed Consent (using the latest approved version of the Informed Consent Form) will then be obtained by means of participant dated signature and dated signature of the individual who presented the study and obtained the Informed Consent. The person who obtained the consent will be suitably qualified and experienced, and have been authorised to do so by the Chief Investigator. A copy of the signed Informed Consent will be given to the participant. The original signed form will be retained in the Investigator study Site File.

**Before any study specific procedures are performed the participant must personally sign and date the latest approved version of the Informed Consent form.**

## Visit 1 - Pre-randomisation phase

Once potential participants have been identified through their responses on the MDQ, they will be invited to the NIHR-CRF for a pre-randomisation phase visit. The pre-randomisation phase visit is expected to last approximately 6 hours and will take place no sooner than 2 weeks prior to randomisation.

At this visit, participants will be presented with a hard copy of the Information Sheet and will be able to discuss the details of the study with the researcher. They will also be given a letter which outlines the details of the services that are available should they feel concerned about their mood. Participants will be given time to consider the information and decide whether they will participate in the study. Written informed consent will then be obtained.

Participants will be screened using the MINI by a researcher or clinician. We anticipate that a subset of participants will meet axis I/II criteria. Regardless of whether they meet axis I/II criteria, the researchers will then ensure that all participants meet the study inclusion and exclusion criteria. Only those that meet the inclusion and exclusion criteria will be eligible to take part in the study (see 7.2 and 7.3).

After assessment of eligibility, participants who meet the study criteria will undergo a research assessment which includes collecting demographic information, clinical history (including duration of illness, previous use of psychotropic medicines, family history of mood disorders, concomitant medication and a physical examination, including being asked to take a pregnancy test. Participants will be asked about past substance use and encouraged to refrain from substance use during the study. The Alcohol Used Disorders Identification Test (AUDIT) will be completed and participants encouraged to limit alcohol consumption to 14 units per week for females and 21 for males. Participants will also be asked to complete short state and trait questionnaires on an IPad, including the Barratt Impulsiveness Scale (BIS), Sleep Condition Indicator, Morningness-Eveningness Questionnaire (MEQ), Maclean Screening Instrument, Affective Lability Scale (short-form), and Affect Intensity Measure. In addition they will also complete screening tools for comorbid borderline personality disorder [Zanarini, 2003] and attention deficit hyperactivity disorder (ADHD) [Adler 2006]. These are all short self-report questionnaires. Participants will be given an e-patch ECG monitor and an actigraphy watch to wear. They will also be given an iPad and instructed on how to complete the daily (PANAS) and weekly (True colours) mood ratings and cognitive tasks.

Blood samples will be taken at this visit and sent for analysis.

A letter will be send to the participants GP informing them that the individual is planning to take part in the study.

## 8.4. Visit 2 - Randomisation

The randomisation visit will take place approximately 2 weeks after study entry.

During this visit eligibility for the randomised phase will be checked including continued consent, absence of contraindications to nicardipine including pregnancy, adherence to cognitive tasks and any current medication. Any difficulties will be explored and, if possible, overcome to enable the participant to continue in the study.

Eligible participants will be entered into the randomised phase (see below 8.5) and provided with study medication. Participants will complete MRI scan and MEG brain scans at this visit prior to taking any study medication. Participants will be asked to arrive for the scans wearing metal free clothes and no make-up. However, changing room facilitates and make up remover are available for participants to prepare on site. The scans will take place at the Oxford centre for Human Brain Activity (OHBA) at the Department of Psychiatry on the Warneford Hospital Site. Participants will be screened for exclusion criteria for scanning again by the radiographer and asked to check for metal. They will also be asked to complete mood ratings before being positioned in the scanner and anatomical and functional scans will be run. They will be asked to rest with their eyes open for part of the time and also to perform simple cognitive tasks involving detecting visual (via screen projection) stimuli and making choices using button-pads or grip-force transducers. The scanners will be operated by a trained radiographer/scanner operator and the task will be administered by a trained researcher. A similar procedure will be followed for the MEG scan. During the MEG scanning eye movements will be tracked. Patients will be in each scanner for no more than 2 hours. Undergoing both scans and completing the cognitive tests will take about 5 hours in total either at a single visit or over 2 visits in close proximity.

At this visit participants will be provided with an e-Patch and asked to wear it for 72 hours, followed by the completion of a sleep questionnaire and a sleep diary. They will be given a portable blood pressure monitor to monitor their blood pressure throughout the two week randomisation phase . Dates for the final study visit and the second MRI and MEG scans will be provided.

A letter will be send to the participants GP informing them that the individual has been randomised to nicardipine or placebo

## 8.5. Visit 3 – final visit

In the second week post randomisation, participants will be invited for the final study visit. This visit is expected to take approximately 7 hours including scanning time (if scans are scheduled on a different day due to scanner availability, this visit will take approximately 2 hours and 20 minutes). At this visit, participants will be asked to complete a battery of neuropsychological tests on a computer. Baseline assessments will be repeated at this visit including weight, pulse, blood pressure, current medication, blood tests and ECG.

Participants will be asked to return all of the devices they had been given at the beginning of the study as outlined in the devices agreement signed at the start of the study (see appendix M) Participants will also be asked to return medication bottles, with any remaining capsules.

Post-treatment MRI scan and MEG scans will be completed under the same conditions as those undertaken pre-treatment.

A letter will be send to the participants GP informing them that the individual has completed the study.

## 8.6. Post-study follow-up

Participants will be contacted within a fortnight of study completion, usually by phone, following cessation of study treatment to review the status of any reported Adverse Events and to check for any unreported AEs.

Please see Appendix B for a schedule of procedures during the study duration.

## Randomisation and allocation concealment

Randomisation will be done in according to an algorithm that minimises on one prognostic factor (see 8.8). Each participant will be randomised to either nicardipine or placebo, with participants, clinicians and researchers involved in study recruitment and assessment visits being blind to allocation.

The computer-generated randomisation schedule will be implemented by a trials manager, based at the University Oxford Department of Psychiatry and who is not involved in the study. A non-deterministic minimisation algorithm will be used to produce treatment groups balanced for important prognostic factors. The first 10 participants will be allocated treatment randomly without minimisation to avoid predictability. Subsequently, the minimisation algorithm will be applied with an allocation ratio that is not fully deterministic: there will be an 80% bias in favour of allocations that minimise the imbalance.

## Minimisation variables

The randomisation algorithm will minimise on gender (M, F).

## Unblinding of treatment

A list of current participants showing their allocation will be maintained by Oxford Health pharmacy staff who are designated unblind.

*Emergency unblinding:* Participants will be given a card to carry to say that they are in the study and may be taking nicardipine or matched placebo. Nicardipine is widely used and therefore any medical doctor attending a study participant who is experiencing a medical emergency will be fully aware of its clinical effects. Participants will be made fully aware of any adverse events they may be likely experience. Participants will also be able to access a consultant-led on-call trials rota which operates 24 hours a day, 7 days a week.

*Non-emergency unblinding:* Allocation of treatments will be recorded on a Randomisation List which will be updated when each new participant enters the randomised phase. The list will be held by unblind staff at the Oxford Health pharmacy. These staff will, on the instructions of the Chief Investigator or delegate, access the list and reveal the allocation to an individual participant.

## Sample Handling

*Blood samples:* Blood samples will be taken using standard phlebotomy techniques. Approximately 50ml will be taken. Tests will include urea and electrolytes, full blood count, LFTs, T4, T3, TSH, leucocyte calcium channel gene expression and studies of calcium signalling. A sample will also be taken to measure calcium level using the InSight™ Electrolyte Analyser located in the NIHR-CRF. Weight/BMI, pulse and blood pressure will also be recorded and an ECG will be performed. Samples for calcium assays will be processed in the NIHR-CRF and analysed using the InSight™ Electrolyte Analyser. Some will also be sent to the pathology laboratory at the John Radcliffe Hospital for a validity check. Assays for IL-6 will be done in the Department of Psychiatry Neurosciences Laboratory. All other blood assays will be done at the John Radcliffe Hospital using standard tests.

Blood samples will be used for genotyping for the CACNA1C risk allele.

Participants will be asked to indicate on the Consent Form whether they are willing for samples to be retained for future research. Samples to be retained for future ethically approved research will be stored in a freezer (-18^o^C) in the University of Oxford Department of Psychiatry, the NIHR CRF or the Department of Pharmacology. At the end of the study, samples from participants who consented to gift them for future research will be linked to key clinical information and then anonymised.

## Discontinuation/Withdrawal of Participants from Study Medication

Each participant has the right to withdraw from the study at any time. In addition, the Researcher may discontinue a participant from the study at any time if s(he) considers it necessary for any reason including:

- Pregnancy
- Ineligibility (either arising during the study or retrospectively having been overlooked at screening)
- Significant protocol deviation
- Significant non-compliance with treatment regimen or study requirements
- An adverse event which requires discontinuation of the study medication or results in inability to continue to comply with study procedures
- Disease progression which requires discontinuation of the study medication or results in inability to continue to comply with study procedures
- Withdrawal of Consent
- Loss to follow up

When a participant stops study medication or withdraws consent, all study data collection will be stopped. Data and samples collected prior to withdrawal will be retained and the reason for withdrawal, if given, will be recorded. Where appropriate, the participant will be asked for a final set of ratings and blood samples and be advised on ongoing treatment, if relevant. If withdrawal is due to an adverse event, the researcher will arrange for follow-up visits or telephone calls until the adverse event has resolved or stabilised. Participants will be asked to return all of the devices they had been given at the beginning of the study as outlined in the devices agreement signed at the start of the study (see appendix M).

## Definition of End of Study

The end of study is the date of the last post-study follow-up of the last participant.

## Description of procedure

***Daily cognitive tasks***. Participants will be asked to complete a battery of neuropsychological tests on a computer. They will also be provided with an iPad with pre-installed apps to enable them to complete short cognitive assessments (Appendix E) plus the PANAS questionnaire twice daily. They will be shown how to use the apps and asked to respond to prompts to complete cognitive assessments for the duration of the study. Data from the apps will be sent wirelessly and electronically to a secured server for analysis.

Participants will be informed that they are able to contact the Research Team during working hours should they have any concerns or if they experience any problems with the iPad or activity monitor(s) (see Appendix F: *Theme 3*).

Imaging interventions: Participants who need optical correction will be asked for details of their current spectacle prescription so that researchers can check whether they will be able to provide MRI/MEG compatible glasses for that prescription (Appendix E).

***Sleep, motor activity and social interactions*.** Participants will be given an activity monitor (GeneActiv wrist-worn accelerometer) and asked to wear it as much as possible from entry into the study until the end of the randomised phase. Participants will be provided with instructions on how to use the monitor and data will be stored on the device itself .

Participants will be given a copy of the Sleep Condition indicator; self-report sleep measure (Espie, 2014), and the Morningness-Eveningness Questionnaire (MEQ) and asked to complete them before wearing the ePatch for 72 hours (see below). Participants will also be asked to keep a sleep diary for the study duration.

***Profile of the calcium signalling system.*** Effects of nicardipine on leucocyte calcium channel expression and calcium signalling will be explored. Participants will be asked to provide blood samples (of approximately 9 teaspoonfuls of blood) pre-calcium treatment at the screening visit and post-calcium treatment at the final visit 2 weeks after randomisation (Appendix G).

***Physiological effects of nicardipine*.** Heart rate and blood pressure will be measured. Participants will also be provided with an e-Patch, shown how to attach it and asked to wear it for a 72-hour period prior to randomisation and 72 hours post-randomisation (Appendix I)**.**

# NICARDIPINE

## Description

Nicardipine will be provided as Cardene SR 30mg capsules. Cardene SR 30mg capsules will be purchased from Oxford Pharmacy Stores. The Department of Psychiatry will (over-)encapsulate Cardene (to mask print on the capsule) and encapsulate matched placebo tablets. Each bottle will be assigned a unique randomisation number to ensure concealment of allocation.

*Nicardipine dose:* Following random allocation (see 8.6), nicardipine/placebo will be given at a dose of 60mg. This will be a stable dose throughout the study period. Participants will be instructed to take the capsules as two separate doses – one 30mg dose after breakfast and one 30mg dose at bedtime*. Nicardipine takes at least 3 days to reach steady state plasma levels.

* Capsules should be taken as near to 12 hours as possible after the previous dose.

## Storage

Nicardipine and placebo will be transferred to the pharmacy at the NIHR-CRF as required. Dispensing will be done by trained staff at the NIHR-CRF. Nicardipine will be stored in a controlled temperature environment and below 25° as per product directions.

## Compliance with Study Medication

Participants will be asked to report adherence to study medication and to return any unused medication. Returned pill counts will be used to provide further tests of adherence.

## Accountability of the Study Medication

All medication will be accounted for by the NIHR-CRF and allocated medication will be recorded on a Drug Allocation Log (DAL) for each participant.

## Concomitant Medication

Participants will be asked to avoid any changes in concomitant medication during the randomised phase as far as possible. Any changes that are made will be recorded.

# SAFETY REPORTING

## Definitions

| **Adverse Event (AE)** | Any untoward medical occurrence in a participant to whom a medicinal product has been administered, including occurrences which are not necessarily caused by or related to that product. |
| --- | --- |
| **Adverse Reaction (AR)** | All adverse events judged by either the Investigator or the Sponsor as having a reasonable suspected causal relationship to an investigational medicinal product qualify as Adverse Reactions. The expression reasonable causal relationship means to convey in general that there is evidence or argument to suggest a causal relationship. |
| **Serious Adverse Event (SAE)** | A serious adverse event is any untoward medical occurrence that:   - results in death - is life-threatening - requires inpatient hospitalisation or prolongation of existing hospitalisation - results in persistent or significant disability/incapacity - consists of a congenital anomaly or birth defect.   Other ‘important medical events’ may also be considered serious if they jeopardise the participant or require an intervention to prevent one of the above consequences.  NOTE: The term "life-threatening" in the definition of "serious" refers to an event in which the participant was at risk of death at the time of the event; it does not refer to an event which hypothetically might have caused death if it were more severe. |
| **Serious Adverse Reaction (SAR)** | An adverse event that is both serious and, in the opinion of the reporting Investigator, believed with reasonable probability to be due to one of the study treatments, based on the information provided. |
| **Suspected Unexpected Serious Adverse Reaction (SUSAR)** | A serious adverse reaction the nature and severity of which is not consistent with the information about the medicinal product in question i.e.:   - in the case of a product with a marketing authorisation, in the summary of product characteristics (SmPC) for that product - in the case of any other investigational medicinal product, in the Investigator’s Brochure (IB) relating to the study in question. |

NB: to avoid confusion or misunderstanding of the difference between the terms “serious” and “severe”, the following note of clarification is provided: “Severe” is often used to describe intensity of a specific event, which may be of relatively minor medical significance. “Seriousness” is the regulatory definition supplied above.

Any pregnancy occurring during the clinical study and the outcome of the pregnancy should be recorded and followed up for congenital abnormality or birth defect, at which point it would fall within the definition of “serious”.

Causality

The relationship of each adverse event to the study medication must be determined by a medically qualified individual according to the following definitions:

*Related:* The adverse event follows a reasonable temporal sequence from study medication administration, cannot reasonably be attributed to any other cause.

*Not Related*: The adverse event is probably produced by the participant’s clinical state or by other modes of therapy administered to the participant.

## Procedures for Recording Adverse Events

Nicardipine has been widely used at the dose planned to be used in the study and therefore has a well-documented safety profile. Symptoms of headache, upset stomach, dizziness or light headedness, excessive tiredness, flushing, numbness, fast heartbeat and muscle cramps, which can occur at therapeutic levels of nicardipine will be monitored at each assessment visit. adverse events (AEs) that are considered related to nicardipine will not therefore be routinely recorded. Recording will only be required for non-serious AEs that are of longer duration or greater severity than would be expected based on the information in the SmPC. For these ARs, the information recorded on the Study-CRF will include a description of the event, the date of onset and end date, severity (1 = mild, 2 = moderate, 3 = severe), assessment of relatedness to study medication, other suspect drug or device and action taken. Follow-up will continue until either resolution or the event is considered stable.

It will be left to the Investigator’s clinical judgment to decide whether or not an AE is of sufficient severity to require the participant’s removal from medication. A participant may also voluntarily withdraw from medication due to what he or she perceives as an intolerable AE. If either of these occurs, the participant will be asked to undergo an end of study assessment and be given appropriate care under medical supervision until symptoms cease, or the condition becomes stable.

## Reporting Procedures for Serious Adverse Events

SAE reporting will cover the period from randomisation and throughout tapering phase until the study nicardipine/placebo has been stopped.

All SAEs must be reported to the Research Team within 24 hours of the researcher becoming aware of the event. The psychiatrist will be asked to complete an SAE Form with the information available at the time of initial reporting and his/her opinion of relatedness and expectedness. S(he) will be asked to supplement this with additional information as it becomes available.

Receipt of an initial SAE report by the OxCaMS RA or NIHR-CRF Lead Nurse for OxCaMS will be acknowledged within 1 working day of receipt and the CI (or delegate) will make an immediate assessment of relatedness and expectedness. The CI will continue to review supplementary information as it is received.

The Chair of the Trial Steering Committee (see 10.6) will be sent details of all SAEs for review by the Committee weekly.

## Expectedness

Expectedness will be determined according to the Summary of Product Characteristics (SmPC) for Cardene SR.

## SUSAR Reporting

A serious adverse event (SAE) occurring to a participant should be reported to the REC that gave a favourable opinion of the study where in the opinion of the Chief Investigator the event was ‘related’ (resulted from administration of any of the research procedures) and ‘unexpected’ in relation to those procedures. Reports of related and unexpected SAEs should be submitted within 15 working days of the Chief Investigator becoming aware of the event, using the HRA [report of serious adverse event](http://www.nres.npsa.nhs.uk/docs/forms/Safety_Report_Form_(non-CTIMPs).doc) form (see HRA website).

See Appendix C for a SAE reporting flow-chart.

## Steering Committee

Although this is an experimental medicine study a Steering Committee will be convened to review the study protocol and to receive progress reports throughout the study. All SAEs will be reported to the committee chair person and SARs and SUSARS will be reported to the committee chair person immediately. The Committee will hold meetings as required (which may be by teleconference) and make recommendations to the CI and/or Sponsor.

The yellow card system will be used to notify the MHRA of any SAEs related to the study medication (<https://yellowcard.mhra.gov.uk/> ).

# ANALYSES

## Description of Statistical Methods

A statistical analysis plan (SAP) will be formulated by the Research Team before the data is analysed. Statistical methods relevant to the nature of the data collected will be employed.

*Cognition:* Data from the cognitive tests will be correlated with the TC and PANAS data. Variability over time will be assessed using the Teager Kaiser energy operator (TKEO) as well as novel approaches to time series data such as rough paths (Lyons et al).

*Neural:* Methods from the fMRIB Statistical Library (FSL) will be used to analyse MRI data. The FSL is a software library containing image analysis and statistical tools for functional, structural and diffusion MRI brain imaging data. MEG data will be analysed using a general linear model (GLM) and ANOVAs. The GLM method is a standard nonlinear beam former used to determine the time course of neuronal activation for each point in a predefined source space.

*Mood:* Mood instability will be quantified using RMSSD. Between group differences and within subject comparisons will be tested using standard parametric / non-parametric tests. We will also apply novel techniques to explore how mood instability changes over the course of the study.

*Behaviour:* Data from the activity monitors, heart rate monitors on motor activation, sleep patterns, and heart rate will be correlated with cognitive data.

*Genetics:* Analysis of changes in gene expression levels pre- and post-calcium treatment will involve comparison of within-subject changes for participant’s allocated nicardipine versus those allocated placebo.

*Physiological:* The results of the ECG and blood pressure tests for participants will be used to explore the effects of nicardipine and placebo on these parameters.

The Number of Participants

The target sample size is 40 randomised participants (20 of whom will be allocated treatment with nicardipine and 20 with placebo).

Power Calculation

The study allows within- and between subject comparisons. As the first study of its kind, there are no directly comparable data for a power calculation. However, recent findings using lithium in a similar experimental medicine imaging paradigm suggests that a sample size of 40 participants will be sufficient to provide more than 90% power to detect difference between groups at a p level of 0.05.

## Criteria for the Termination of the Study

The study is not designed to evaluate the efficacy of nicardipine on mood symptoms and therefore stopping rules based on criteria for demonstration of a robust effect or clear lack of effect are not appropriate.

## Procedure for Accounting for Missing, Unused, and Spurious Data.

The majority of the outcome measures are provided by participants via TC, PANAS, daily cognitive tests and the carrying of activity monitors and smartphones. Adherence to these rating will be reviewed regularly and any concerns about missing ratings will be addressed with the participant at the next appointment. In addition to this, where possible, participants who do not complete weekly TC ratings will be contacted and asked to complete the ratings over the phone.

## Inclusion in Analysis

The main statistical analyses will be per protocol with data for all participants being included up to the point where they stop study treatment or complete the randomised phase.

# DATA MANAGEMENT

## Source Data

Source documents are where data are first recorded, and from which participants’ Study-CRF data are obtained. These include, but are not limited to clinical and office charts, laboratory and pharmacy records, diaries, and correspondence.

Study-CRF entries will be considered source data if the Study-CRF is the site of the original recording (i.e. where there is no prior written or electronic record of data). All documents will be stored safely in confidential conditions. On all Study-CRFs except the Informed Consent Form, the participant will be referred to by non-personal identifiable information not by name.

## Access to Data

Direct access will be granted to authorised representatives from the Sponsor, Oxford University, and the regulatory authorities to permit study-related monitoring, audits and inspections.

## Data Recording and Record Keeping

All study data will be entered on to paper CRFs and/or a validated database for which Standard Operating Procedures are in place. Participants will be identified by non-personal identifiable information in any study specific database.

Data will be retained for 5 years after last visit last patient (LVLP).

# QUALITY ASSURANCE PROCEDURES

The study will be conducted in accordance with the current approved protocol, GCP, relevant regulations.

Reports of SAEs will be reviewed by the Steering Committee (See 10.7)

# SERIOUS BREACHES

In the event that a serious breach is suspected the Sponsor must be contacted within 1 working day. In collaboration with the C.I., the serious breach will be reviewed by the Sponsor and, if appropriate, the Sponsor will report it to the REC committee.

# ETHICAL AND REGULATORY CONSIDERATIONS

## Declaration of Helsinki

The CI will ensure that the study is conducted in accordance with the principles of the Declaration of Helsinki.

## Guidelines for Good Clinical Practice (GCP)

The CI will ensure that the study is conducted in full conformity with relevant regulations and with the Guidelines for Good Clinical Practice.

## Approvals

The protocol, informed consent form, participant information sheet and any proposed advertising material will be submitted to an appropriate Research Ethics Committee (REC), and HRA for written approval.

The Investigator will submit and, where necessary, obtain approval from the above parties for all substantial amendments to the original approved documents.

## Reporting

The CI shall submit once a year throughout the study, or on request, an Annual Progress report to the REC Committee, HRA, host organisation and Sponsor. In addition, an End of Study notification and final report will be submitted to the same parties.

## Participant Confidentiality

The study staff will ensure that the participants’ anonymity is maintained. The participants will be identified only by non-personal identifiable information on the Study-CRFs and any study-specific electronic database. Contact details for all participants will be stored separately from their data. This will be held in a locked cupboard at the NIHR-CRF and destroyed once they are no longer required for the follow-up call. All documents will be stored securely and only accessible by the Research Team and authorised personnel. The study will comply with the General Data Protection Regulation and Data Protection Act 2018, which requires data to be anonymised as soon as it is practical to do so.

## Expenses and Benefits

Participants completing the randomised phase will be given the choice of keeping the iPad or being reimbursed £50 per study visit attended. If brain scans have to be scheduled on a different day to study visits, participants will be reimbursed £25 for study visit and £25 for the brain scan visit. All participants will be able to claim reasonable travel expenses, including costs of parking for research-related visits and, for lengthy visits, will be provided with refreshments.

## Other Ethical Considerations

*Inclusion of placebo arm:* Patients with current mood instability will have a 50% chance of being allocated placebo with no active psychotropic medicine. This should not have adverse effects on patients because anyone requiring urgent treatment for mood symptoms would not eligible for the study and any participant whose mood deteriorates during the study will be encouraged to contact their GP or psychiatrist for appropriate treatment. Participants will be provided with a study treatment card which will give brief details of the study as well as contact numbers for the study team.

*Participant burden:* The study involves a number of different procedures but the time and effort required from participants to complete each of these has been kept to a minimum.

The main element of participant burden arising from the study is the time required for visits and to complete tasks.

*Visits:* The study involves a total of 3 (possibly 5) visits. The three necessary visits are for the study screening, study randomisation and the final study visit. These visits each take several hours. If MRI and MEG scans cannot be booked on the same days as these study visits, participants may have to attend a further 2 shorter visits to complete study scans. Patients will be advised of this in advance and will be provided with refreshments and breaks.

*Time:* Completion of PANAS and cognitive tasks will take about 15 minutes each day. The data collection for the continuous measures requires little input from participants apart from wearing/carrying the activity monitor and smart device neither of which should be a hindrance in any way.

Participants will also be asked to complete weekly True Colours ratings of depressive and manic symptoms.

*Genetic testing:* Participants will not be informed about the results of the genetic testing as the CACNA1C gene does not have any direct implications for health or wellbeing.

*Scans:* Completing both scans and the neuropsychological test battery will take about 5 hours. Whenever possible, participants will be able to choose whether to have the scans on the same day or on different days. Scanner operators will ensure that participants are comfortable and relaxed and will provide them with an emergency button that the participant can use at any time to indicate that they want to be released.

*Incidental findings:* Incidental findings. Delete current paragraph and replace with: ‘We anticipate that a subset of participants will meet criteria for an axis I/II disorder. At the discretion of the investigator, and with the permission of the participant, their GP will be informed, and the participant advised to seek advice about treatment’. Participants will also be referred to their GP for any unexpected health issues.

In the unlikely event of seeing any structural abnormalities on an MRI scan, the scan will be checked by a clinical specialist. If the specialist feels that the abnormality was medically important, they will discuss the implications with the participant and arrange for further investigations as necessary. Participants will not be informed unless the doctor considers the finding has clear implications for their current or future health. It is important to note that scans are not carried out for diagnostic purposes, and therefore the scans are not a substitute for a clinical appointment. Rather, the scans are intended for research purposes only.

*Intrusiveness of data gathering and nature of data gathered*

Participants will receive emails or text messages asking them to complete self-ratings but they will be able to choose the time of day when these prompts are sent. Participants who do not respond to an email or text message prompt will receive a single reminder the following day and may be contacted and asked to complete the ratings over the phone.

*Ability to consent:* Patients who are not able to give informed consent are not eligible for the study.

*Involvement of vulnerable participants:* The research is being conducted within the Oxford Health NHS Foundation Trust and the Department of Psychiatry at the University of Oxford and more junior researchers will be able to consult a study psychiatrist should they have any concerns about the mental state of a participant. If a participant were to become unwell during the study permission would be sought to contact their care team (GP or psychiatrist). It will be made clear to all participants that taking part in the study is in addition to routine care and that, if they feel unwell during the course of the study, they should contact their GP or care team.

# FINANCE AND INSURANCE

## Funding

The study is being funded from the CONBRIO (Collaborative Network for Bipolar Research to Improve Outcomes) Wellcome Trust Strategic Award and the NIHR Oxford Health Biomedical Research Centre. CONBRIO is a programme of translational research intended to transform the understanding and treatment of bipolar disorder.

## Insurance

The University has a specialist insurance policy in place which would operate in the event of any participant suffering harm as a result of their involvement in the research (Newline Underwriting Management Ltd, at Lloyd’s of London).

# PUBLICATION POLICY

The main results of the study will be published together in an appropriate journal and made available to participants via the Department of Psychiatry website. Additional results from the individual themes will be published in topic-specific journals.

# REFERENCES

Adler LA, Spencer T, Faraone SV et al. Validity of pilot Adult ADHD Self-Report Scale (ASRS) to rate adult ADHD symptoms. Annals of Clinical Psychiatry 2006;18(3):145-148.

Albert U, DeCori D, Aguglia A et al. Lithium-associated hyperparathyroidism and hypercalcaemia: A case-control cross-sectional study. J Affect Disord. 2013;151:786-790.

Altman EG, Hedeker D, Peterson JL et al. The Altman Self-Rating Mania Scale. Biological Pscyhiatry. 1997;42(10):948-985.

Angst  J, Gamma  A, Sellaro  R et al. Recurrence of bipolar disorders and major depression: a life-long perspective. Eur Arch Psychiatry Clin Neurosci. 2003;253,(5):236- 240.

Bassett D. A literature review of heart rate variability in depressive and bipolar disorders. Aust N Z J Psychiatry December 23, 2015 [E-Pub ahead of print] doi:

10.1177/0004867415622689

Bauer M, Grof P, Gyulai L et al. Using technology to improve longitudinal studies: self-reporting with ChronoRecord in bipolar disorder. 2004;60(1):67-74.

Bedford JJ, Weggery S, Ellis G et al. Lithium-induced Nephrogenic Diabetes Insipidus: Renal effects of amiloride. Clin J Am Xoc Nephrol. 2008; 3:1324-1331.

Bopp JM, Miklowitz DM, Goodwin GM et al. The longitudinal course of bipolar disorder as revealed through weekly text-messaging. Bipolar Disord. 2010;:327-334.

Bourne C, Aydemir Ö, Balanzá-Martínez V, Bora E, Brissos S, Cavanagh JT et al. Neuropsychological testing of cognitive impairment in euthymic bipolar disorder: an individual patient data meta-analysis. *Acta Psychiatr Scand* 2013; **128:** 149-162.

Broome MR, Saunders KEA, Harrison PJ, Marwaha S. Mood instability: significance, definition and measurement. British Journal of Psychiatry 2015; 207(4) 283-285.

Byrne EM, Gehrman PR, Medland SE, Nyholt DR, Heath AC, Madden PA et al. A genome-wide association study of sleep habits and insomnia. *Am J Med Genet B Neuropsychiatr Genet* 2013; **162B**: 439-451

Chen HM, DeLong CJ, Bame M, Rajapakse I, Herron TJ, McInnis MG et al. Transcripts involved in calcium signaling and telencephalic neuronal fate are altered in induced pluripotent stem cells from bipolar disorder patients. *Transl Psychiatry* 2014; **4**: e375.

Cipriani A, Saunders KEA, Attenburrow MJ, Sefaniak J, Panchal P, Stockton S, Lane TA, Tunbridge EM, Geddes JR, Harrison PJ. A systematic review of calcium channel antagonists in bipolar disorder and some considerations for their future development. Accepted for publication in Molecular Psychiatry 2016

Craddock N, Sklar P. Genetics of bipolar disorder. *Lancet* 2013; **381**: 1654-1662.

Espie CA, Kyle SD, Hames P, Gardani M, Fleming L, Cape J. The Sleep Condition Indicator: a clinical screening tool to evaluate insomnia disorder. BMJ Open. 2014. 4:e004183.

doi:10.1136/bmjopen-2013-004183

Ferreira MA, O'Donovan MC, Meng YA, Jones IR, Ruderfer DM, Jones L et al. Collaborative genome-wide association analysis supports a role for ANK3 and CACNA1C in bipolar disorder. *Nat Genet* 2008; **40:** 1056-1058.

Fiorentino A, O'Brien NL, Locke DP, McQuillin A, Jarram A, Anjorin A et al. Analysis of ANK3 and CACNA1C variants identified in bipolar disorder whole genome sequence data. *Bipolar Disord* 2014; **16**: 583-591.

Geddes JR, Miklowitz DM. Treatment of bipolar disorder. The Lancet. 2013;381:1672-1682.

Gelenberg AJ, Thase ME, Meyer RE et al. The history and current state of antidepressant clinical study design: A call to action for proof-of-concept studies. J Clin Psych. 2008;69:1513-1528.

Hahn CG, Gomez G, Restrepo D, Friedman E, Josiassen R, Pribitkin EA et al. Aberrant intracellular calcium signaling in olfactory neurons from patients with bipolar disorder. *Am J Psychiatry* 2005; **162**: 616-618.

Harvey, A.G., et al., Sleep-related functioning in euthymic patients with bipolar disorder, patients with insomnia, and subjects without sleep problems. Am J Psychiatry, 2005. 162(1): p. 50-7.

insomnia, and subjects without sleep problems. Am J Psychiatry, 2005. 162(1): p. 50-7.

Harvey AG. Sleep and circadian rhythms in bipolar disorder: seeking synchrony, harmony, and regulation. *Am J Psychiatry* 2008; **165**: 820-9.

Heck A, Fastenrath M, Coynel D, Auschra B, Bickel H, Freytag V et al. Genetic Analysis of Association Between Calcium Signaling and Hippocampal Activation, Memory Performance in the Young and Old, and Risk for Sporadic Alzheimer Disease. *JAMA Psychiatry* 2015; **72**: 1029-1036.

Jimerson DC, Post RM, Carman JS, van Kammen DP, Wood JH, Goodwin FK et al. CSF calcium: clinical correlates in affective illness and schizophrenia. *Biol Psychiatry* 1979; **14**: 37-51

Judd LL, Akiskal HS, Schettler PJ et al. The long-term natural history of the weekly symptomatic status of bipolar I disorder. Archives of General Psychiatry. 2002;59(6):530-537.

Judd LL, Akiskal HS, Schettler PJ et al. A prospective investigation of the natural history of the long-term weekly symptomatic status of bipolar II disorder. Archives of General Psychiatry. 2003; 60(3): 261-269.

Lancaster TM, Heerey EA, Mantripragada K, Linden DE. CACNA1C risk variant affects reward responsiveness in healthy individuals. *Transl Psychiatry* 2014; **4**: e461.

McCarthy MJ, Le Roux MJ, Wei H, Beesley S, Kelsoe JR, Welsh DK. Calcium channel genes associated with bipolar disorder modulate lithium's amplification of circadian rhythms. *Neuropharmacol* 2016; **101**: 439-448.

McDonald KC, Saunders KE, Geddes, JR, Sleep problems and suicide associated with mood instability in the Adult Psychiatric Morbidity Survey, 2007. Aust N Z J Psychiatry 2017;51(8):822-828.

McKnight R, Adida M, Budge K et al. Lithium toxicity profile: a systematic review and meta-analysis. The Lancet. 2012;379:721-728.

Merikangas K, Jin R, Jian-Ping He et al. Prevalence and correlates of bipolar spectrum disorder in the World Mental Health Survey Initiative. Arch Gen Psychiatry. 2011;68(3):241-251.

Mertens J, Wang QW, Kim Y, Yu DX, Pham S, Yang B et al. Differential responses to lithium in hyperexcitable neurons from patients with bipolar disorder. *Nature* 2015; **527**: 95-99.

Parsons MJ, Lester KJ, Barclay NL, Nolan PM, Eley TC, Gregory AM. Replication of Genome-Wide Association Studies (GWAS) loci for sleep in the British G1219 cohort. *Am J Med Genet B Neuropsychiatr Genet* 2013; **162B**: 431-8.

Petcharunpaisan S, Ramalho J, Castillo M. Arterial spin labelling in neuroimaging. World J Radiol. 2010 Oct 28; 2(10): 384–398.

Rush AJ, Trivedi MH, Ibrahim HM et al. The 16-item Quick Inventory of Depressive Symptomatology (QIDS), clinician rating (QIDS_C) and self-report (QIDS-SR): a psychometric evaluation in patients with chronic bipolar depression. Biological Psychiatry. 2003;54:573-578.

Strohmaier J, Amelang M, Hothorn LA, Witt SH, Nieratschker V, Gerhard D et al. The psychiatric vulnerability gene CACNA1C and its sex-specific relationship with personality traits, resilience factors and depressive symptoms in the general population. *Mol Psychiatry* 2013; **18**: 607-513.

Thompson ER, Development and Validation of an internationally reliable short-form of the Positive and Negative Affect Schedule (PANAS). Journal of Cross-Cultural Psychology. 2007;38(2):227-242.

Wendt ER, Ferry H, Greaves DR et al. Ratiometric Analysis of Fura Red by Flow Cytometry: A Technique for Monitoring Intracellular Calcium Flux in Primary Cell Subsets. PLoS ONE 2014; 10 (4): e0119532.

Yoshimizu T, Pan JQ, Mungenast AE, Madison JM, Su S, Ketterman J et al. Functional implications of a psychiatric risk variant within CACNA1C in induced human neurons. *Mol Psychiatry* 2015; **20**: 162-169.

Zanarini MC, Vujanovic AA, Parachini EA et al. A screening measure for BPD: The McLean Screening Instrument for Borderline Personality Disorder (MSI-BPD). Personal Disord 2003; 17: 568– 73.

# Appendix A: Participant pathway

**Visit 2- Randomisation**

- Check on adverse events

**Follow-up Call**

- Confirmation of willingness to continue in the study
- Eligibility check –adherence to rating scales
- Randomisation:

If eligible:

**Allocated nicardipine/placebo**

- Pre-treatment brain scans
- Provided with second e-Patch heart rate monitor
- Provided with portable blood pressure monitor
- Blood samples taken, weight, blood pressure, pulse and ECG repeated.
- Cognitive tests completed
- Post-treatment brain scans
- Device(s) returned.
- Checks for adverse events
- Informed Consent
- Eligibility assessment by using the MINI and completing a battery of cognitive tests
- Medical history and pregnancy test
- Weight, blood pressure and pulse recorded
- Baseline cognitive tasks and ECG completed
- iPad and activity monitor set-up and provided
- Weekly and daily self-reporting started.
- Blood samples taken
- Provided with e-Patch heart rate monitor and sleep diary for a 72-hour period prior to randomisation

**4 weeks**

Carry the GeneActiv wrist worn activity monitor and complete questionnaires and cognitive tasks on an iPad - daily

True Colours ratings of mood –weekly

# Appendix B: Schedule of Procedures

| Attendance number | **1** | **2** | **3** | **4** |
| --- | --- | --- | --- | --- |
| Timeline (days) | **0** | **14** | **28** |  |
| Window (days) |  | **±2** | **±2** |  |
| Informed consent | ✓ |  |  |  |
| Demographics | ✓ |  |  |  |
| Diagnosis (MINI) | ✓ |  |  |  |
| Medical history | ✓ |  |  |  |
| Physical examination | ✓ | ✓ |  |  |
| ECG | ✓ |  | ✓ |  |
| Vital signs and BMI | ✓ | ✓ |  |  |
| Eligibility assessment | ✓ | ✓ |  |  |
| Follow-up call |  |  |  | ✓ |
| Personality disorder screen | ✓ |  |  |  |
| ADHD screen | ✓ |  |  |  |
|  |  |  |  |  |
| Neuropsychological tests | ✓ |  | ✓ |  |
| Randomisation |  | ✓ |  |  |
| True Colours | **✓** Weekly | | |  |
| PANAS | **✓** Daily | | |  |
| Cognitive tests | **✓** Daily | | |  |
| Carry activity monitor | **✓** Daily (with regular data downloads) | | |  |
| Concomitant medications | ✓ | ✓ | ✓ |  |
| Dispensing study drugs and pill count |  | ✓ | ✓ |  |
| Adverse Event check |  | ✓ | ✓ | ✓ |
| MEG |  | ✓* | ✓ |  |
| MRI |  | ✓* | ✓ |  |
| Sleep questionnaire | ✓ | ✓ | ✓ |  |
| Sleep Diary card commenced | ✓ | ✓ |  |  |
| Sleep Diary card completed & returned |  |  | ✓ |  |
| ePatch** | ✓ | ✓ |  |  |
| Portable blood pressure monitor*** |  | ✓ |  |  |
| Blood tests | ✓ |  | ✓ |  |
| Devices returned |  |  | ✓ |  |

- *MEG and MRI scan prior to taking any study medication.
- ** Wear for 72 hours
- *** Participants to record blood pressure once daily throughout two-week randomisation phase

# Appendix C: SAE Reporting Flow Chart

**Adverse event**

**Adverse Event**

**No further action**

**Related to IMP?**

**Serious? (See box)**

**Expedited report to REC and Sponsor within:
 - 7 days if fatal/life-threatening, - 15 days if not**

**Yellow card system report**

**Ongoing Safety Review
by Steering Committee**

**Adverse Reaction**

**Serious Adverse Event**: any untoward medical occurrence that:

- results in death
- is life-threatening
- requires inpatient hospitalisation or prolongation of existing hospitalisation
- results in persistent or significant disability/incapacity
- consists of a congenital anomaly or birth defect.

Other ‘important medical events’ may also be considered serious if they jeopardise the participant or require an intervention to prevent

**Timeframe:** Must be reported to Research Team within 24 hours of becoming aware of SAE

**Inform Investigators**

**SUSAR**

**Expected?
(See SmPC)**

**Serious Adverse Reaction**

**Serious Adverse Event**

**Related to IMP?**

**Consistent with SmPC?**

# Appendix D: Rating scales

**Rating Scales**

Weekly mood ratings for the study will be captured on four scales, the Quick Inventory of Depressive Symptomatology (QIDS-SR_16_), the Altman Self-Rating Mania Scale (ASRM), the Generalised Anxiety Disorder Questionnaire (GAD-7) and the Quality of Life questionnaire (EQ-5D).

*QIDS-SR_16_*: The QIDS-SR_16_ is a 16-item self-report questionnaire that covers 9 key symptoms of depression (Rush, 2003). The scale is designed to assess both severity of depression and change in depressive symptoms over time.

Participants are instructed to score each item according to the description that best describes how they have been over the past 7 days. Each of the symptoms is scored on a 4-point scale (0 – 3) giving a maximum score of 27.

*ASRM:* The ASRM is a 5-item self-report questionnaire that assesses severity of and change in manic symptoms (Altman 1997).

Participants are instructed to score each item according to the description that best describes how they have been over the past 7 days. Each of the symptoms is scored on a 5-point scale (0 – 4) giving a maximum score of 20.

*GAD-7:* The GAD-7 is a 7-item questionnaire that assesses the frequency of anxious thoughts and behaviours (Spitzer et al., 2006).

Participants are instructed to score each item according to the description that best describes how they have been over the past 7 days. Each of the symptoms is scored on a 4-point scale (0 – 3) giving a maximum score of 21.

*EQ-5D*: The EQ-5D is a general health questionnaire assessing mobility, activity, self-care, the presence of pain or discomfort and anxiety or depression, and overall health state.

Participants are instructed to indicate whether they have no problems, some problems or are significantly affected by the above categories over the past 7 days. They are also asked to indicate on a scale their health state from 0 – worst imaginable state to 100 – best imaginable state.

**PANAS**

PANAS (Thompson, 2007) will be used to measure day-to-day mood variations which will be used as a covariate in the analysis of the daily cognitive tasks. Participants will be prompted to rate the extent to which 10 mood descriptors best describe their mood over that day. The ten descriptors are upset, hostile, alert, ashamed, inspired, nervous, determined, attentive, afraid, and active. Each is rated on a 5-point scale from never to always.

# Appendix E: Theme 2 Cognitive tests and neural dynamics

**Neuropsychological tests at baseline and in week 4.**

At the screening and scanning appointments, participants will be asked to complete a battery of cognitive tasks related to emotional processing, working memory, attention and behavioural inhibition which participants complete on a computer. In addition they will be tested for IQ.

**Daily cognitive tasks**

Participants will be provided with an iPad on which they will complete daily cognitive tasks via the True Colours System.

Thorough details of the cognitive tasks will be provided in the participant information sheet.

The tasks should not take longer than 10 minutes to complete and will tap into working memory, learning and attention processes.

**Scans**

Both scans will record resting state activity and brain activity in response to cognitive tasks. MRI and MEG scans will take place at the Oxford centre for Human Brain Activity (OHBA) at the Department of Psychiatry Warneford Hospital Site.

**MRI**

Once contraindications to magnetic resonance imaging are excluded by use of the facility’s screening forms, the risks of undergoing a scan are minimal. A trained scanner operator or radiographer will go through a list of possible risks with the participant before scanning. The MRI scanner consists of a large powerful magnet. Magnetic resonance imaging uses no ionising radiation. There are, however, potential hazards associated with MRI and the scanning of participants including the presence of surgical implants, participants’ clothing, jewellery (such as body piercings) bodily habitus, or medical conditions. A comprehensive list of potential risks has been compiled, and the participant should be checked against this by the operator, prior to entering the controlled areas of the MRI scanners. During the actual scanning procedure, the scanner produces loud banging noises and the participant will be given suitable hearing protection (earplugs and protective headphones). There is a small mirror that will allow them to see out of the scanner. During the experiment, the participant will be able to communicate with the operator in the control room. In addition, they will be given a call button, which allows them to alert the operator at any time. People with a history of claustrophobia may be excluded from participation in the study. All participants will still be introduced carefully to the scanner and allowed to leave at any stage, should they wish to do so. Once in the scanner, participants will be able to indicate immediately if they wish the scanning to cease by pressing a call button in their hands.

*MRI measurements*

- T1- weighted image: Structural scan to provide anatomical information
- T2-weighted image: FLAIR scan to provide information about white matter hyperintensities (WMH) lesions
- Diffusion tensor imaging (DTI): scan to provide information about white matter properties and fibre tracts
- Resting state scan: eyes open, participants will be asked to stay in the scanner and think of nothing in particular
- Task based activation scan

.

**MEG**

MEG is a silent, non-invasive brain imaging technique that measures the magnetic fields produced by nerve cells. During the MEG scanning eye movements will be tracked.

The MEG system contains very sensitive detectors arranged around a helmet shaped hollow. Brain activity is measured from a participant as they sit with their head inside this hollow. Because the magnetic signals produced by brain activity are tiny compared to those produced by the earth and electrical equipment, the scanner is in a specially built room that keeps out magnetic fields from the environment. MEG does not generate any magnetic fields and does not involve any ionising radiation. There are no known risks associated with MEG.

MEG is a very sensitive technique and measurements can be affected by metal in the room. Participants will be asked to remove metallic objects that they are carrying or wearing, for example, jewellery, body piercings, removable dental braces and clothing with metal parts. Participants with metal in their body (e.g. plates, dental work, pacemakers) will discuss this with the researcher in advance.

Those who wear glasses will inform the researcher in advance and they may be given special non-metallic glasses to wear. In some cases it may not be possible to scan the participant.

Before the MEG scan, the researcher will attach sensors to the participant’s wrists to measure their heartbeat and around their eyes to measure eye movements. The researcher will also place small coils on the participant’s forehead and above their ears to record their head position in the scanner. Participants can ask the researcher to stop at any time.

*MEG measurements*

Resting state scan

Task based activation scan

# Appendix F: Theme 3 Sleep, motor activity and social interactions

A maximum of two activity monitors will be given to participants for the duration of the study to measure levels of physical activity and sleep patterns that can be correlated with True Colours mood ratings (Theme 1) and the cognitive ratings and PANAS (Theme 2).

**Activity monitor**

| 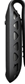 | **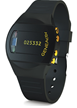** | Activity monitors (examples shown) make a number of measures of activity. Raw data can be used to provide information about frequency and amplitude of movements during daytime activity; categorization of activity types; as well as duration, timing, and quality of sleep. |
| --- | --- | --- |
| 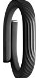 | |  |

*Carrying the activity monitor:* Activity monitors are designed to be worn in different ways. Typically monitors can be carried in a pocket, clipped to clothing, or worn on the wrist. Participants will be encouraged to wear the activity monitors continually, except when the device are not waterproof, where participants will be asked to take the device off when bathing or showering.

*Data transfer:* GeneActiv wrist-worn accelerometers store data on an internal memory card, and data can be downloaded during research appointments.

*Viewing data*: Participants are unable to view data whilst they are being collected but can do so if they wish when the device is returned and data are downloaded (as above).

*Charging the monitor:* The GeneActiv devices do not require charging by the participant.

# Appendix G: Theme 4 Profile of the calcium signalling system

Changes in gene expression levels will be measured from pre- and post-calcium treatment blood samples from participants.

**Assays**

Calcium channel subunit expression will be measured in blood samples. Leucocyte calcium signalling will be measured using established assays (Wendt 2015).

**Nicardipine card and dose instructions**

Participants will be provided with:

- an OxCaMS card indicating that they are either taking Cardene or matched placebo. The card will record current dose and will be replaced if the dose is changed
- information about nicardipine including details of adverse effects which, if experienced, would require immediate medical attention
- a record sheet with key physiological information collected at the screening and randomisation visits.

# Appendix I: ePatches

The ePatch has been designed to provide reliable high quality continuous ECG monitoring for long periods of time without any patient discomfort or impairment of normal daily life activities.

Following recording data can be downloaded via a micro USB and can be visualised and analysed in the same way as ECG’s recorded on traditional equipment.

ePatches consist of a re-usable sensor with a single use electrode. Each electrode provides continuous ECG recording for a 72-hour period

Participants will be asked to wear ePatches for two 72-hour periods during OxCaMS. The first period will be during the pre-randomisation phase. The second period will be after the randomisation visit and before the 4-week visit. At the start of each recording period they will be asked to remain seated for 10 minutes to enable a resting ECG to be recorded.

| **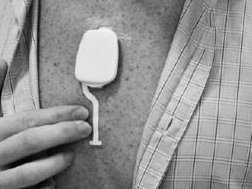** | ePatches have an adhesive back which sticks to (hair-free) skin and are placed in the centre of the chest underneath clothing. They can be worn under normal clothing and are shower proof. |
| --- | --- |

# Appendix J: Screening questionnaires

### Barratt Impulsiveness Scale (BIS-11)


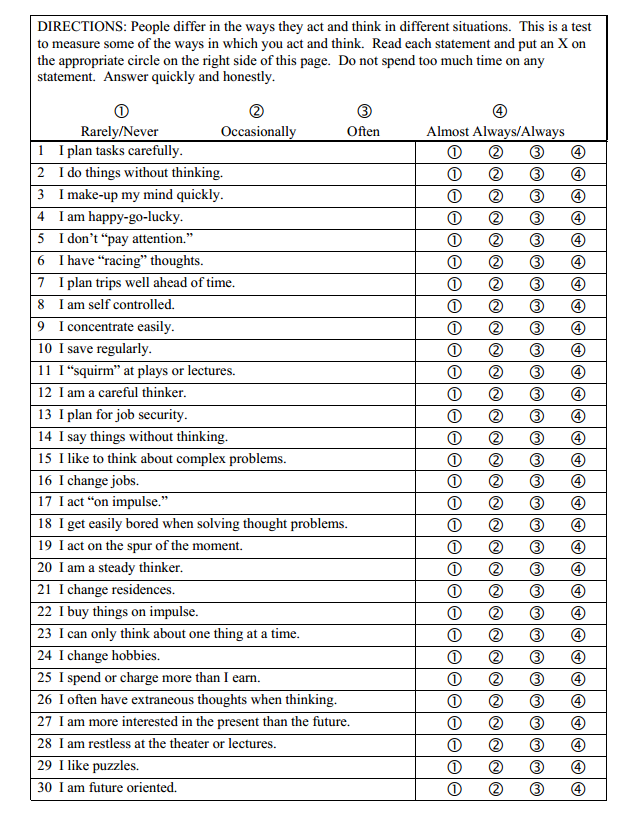


###

### Sleep Condition Indicator (SCI-R)


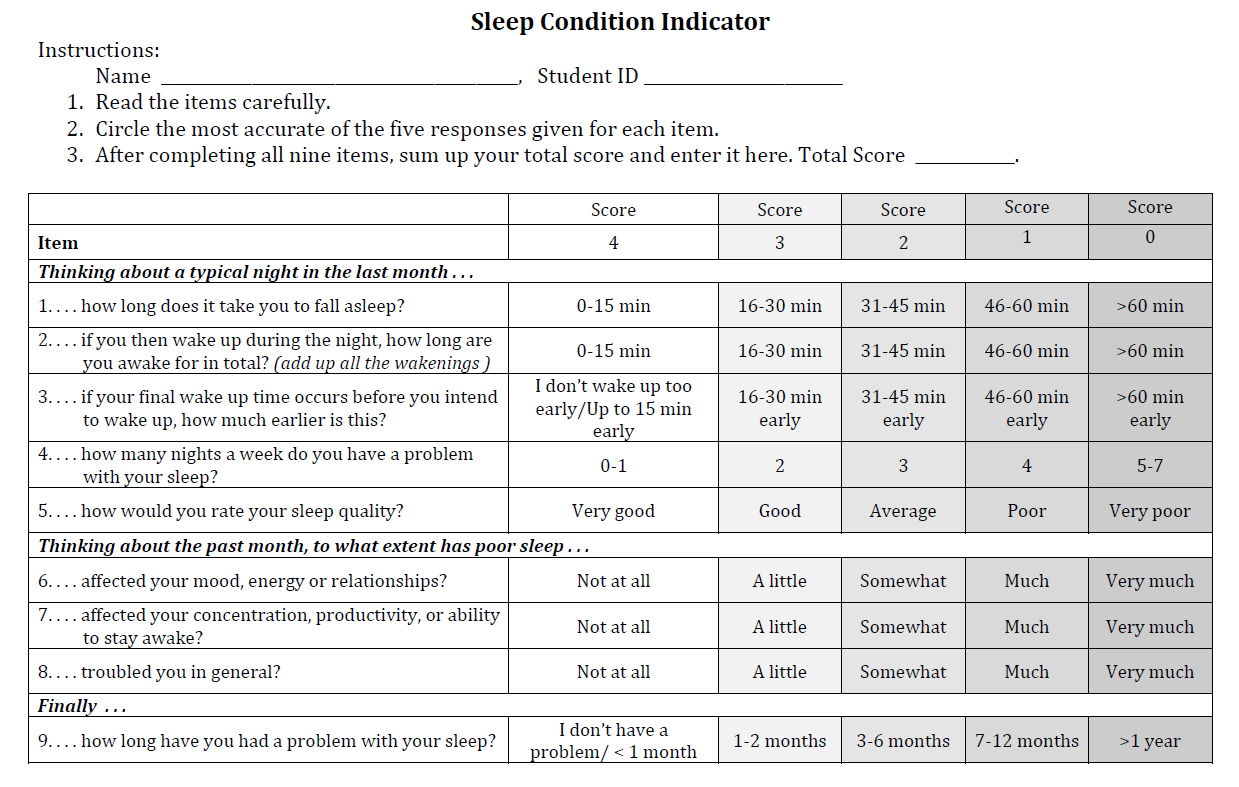


### Maclean Screening Instrument (MSI-BPD)


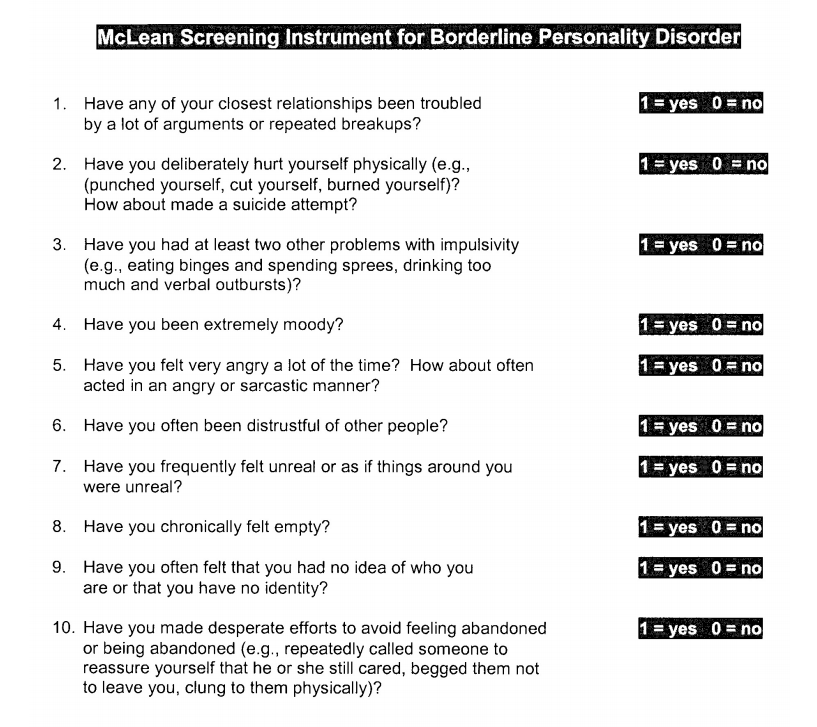


### Affective Lability Scale – Short Form (ALS-SF)


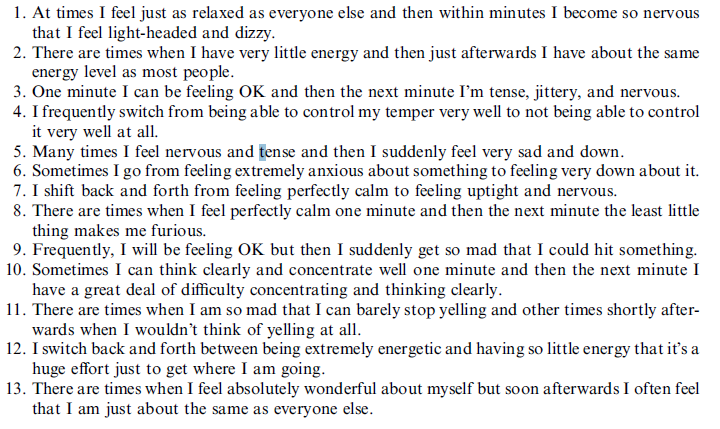

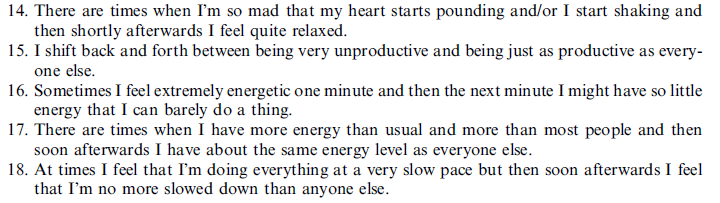


###
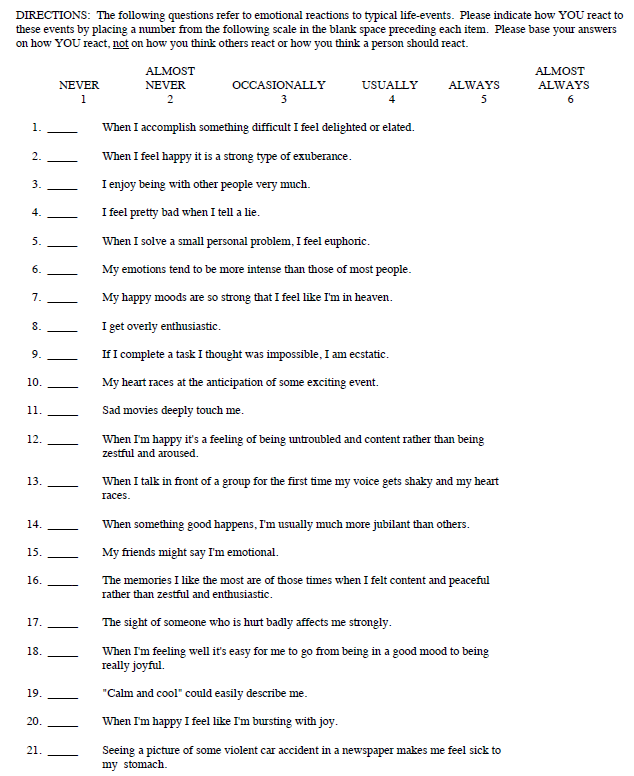
Affect Intensity Measure (AIM)


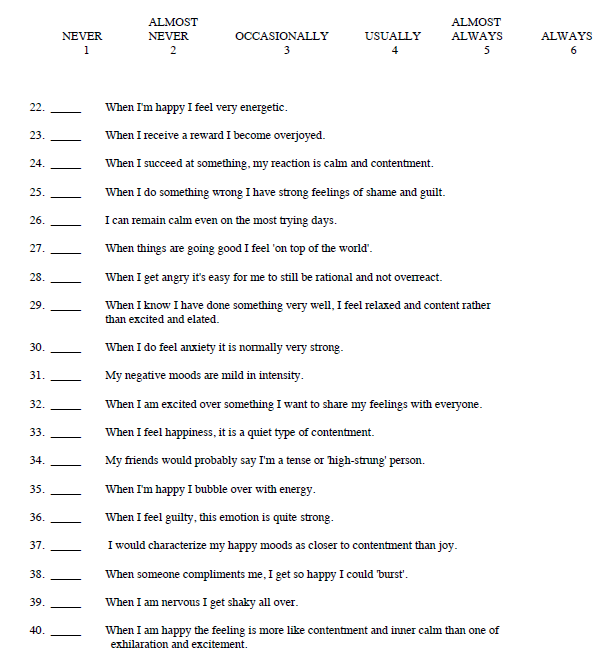


# Appendix K: Collaborator agreement

**OxCaMS**

OxCaMS: The Oxford Study of Calcium Channel Antagonism, Cognition, Mood Instability and Sleep.

Collaborator Agreement

I have read this protocol and agree to abide by all provisions set forth therein.

I agree to comply with the International Conference on Harmonisation Tripartite Guideline on Good Clinical Practice.

Signature

Name

Date

Name of site / institution

Email

Role in OxCaMS

Confidentiality Statement

*The information contained in this document must not be used for any purpose other than the evaluation or conduct of the clinical investigation without the prior written consent of Professor Harrison.*

***The original signed copy of this page should be sent the OxCaMS Research Team***

# Appendix M: Devices agreement

Participant ID____________________________

I understand that all devices must be returned to the clinical research facility or the department of psychiatry at the end of the study period or if I decide to withdraw from the study. I am aware that if I fail to return the devices they will be reported as stolen to the police.

Signed_______________________ Date___/___/___

# Appendix L: Amendment History

| **Amendment No.** | **Protocol Version No.** | **Date issued** | **Author(s) of changes** | **Details of Changes made** |
| --- | --- | --- | --- | --- |
| 1 | V1.3 | 20/08/2017 | Dr Kate Saunders | - Addition of a further secondary objective in order to maximise the use of the data that we are collecting. This additional objective relates to the mood measurements we are taking throughout the study. Specifically we would like to explore whether there are any changes in the stability of mood (as opposed to mean mood scores) associated with nicardipine. These changes are applicable to IRAS form A58 and pages 9 and 13 of the protocol (v1.3)   Additional minor changes:  1. New members of the team:  Dr Lucy Potter, Priyanka Panchal (will have participant contact)  Prof. Elizabeth Tunbridge, Arne Mould (no participant contact - lab-based)  Simon Bond, Alex Irvine (no participant contact - IT support)  2. Additional funding source listed. Protocol page 1 V1.3  3. Change to the start and end date of the study as there have been delays to the study commencing. The overall duration remains the same. Protocol page 7 V1.3  4. Change to the design of the randomisation schedule. This will now be handled by an independent trials manager. Protocol page 19 V1.3  5. Option made available for participants to contact the out of hours rota for trials in the event of a study related query. Protocol page 19 V1.3  6. Minor typographical changes to the protocol to correct spelling and formatting errors Protocol V1.3  7. Inclusion of a 10-minute rest period at the start of the e-patch recording. Overall recording time remains unchanged. Protocol page 44 V1.2  8. Removal of a paragraph from the PIS – this data collection is not included in the protocol and should not be in the PIS. PIS page 4 V1.2  9. Change to the wording from ‘your doctor’ to ‘the study doctor’. PIS page 6 V1.2 |
| 2. | V1.4 | 12/12/2017 | KS/LA/LC/PH | See attached |
| 3. | V1.5 | 13/05/2018 | KS/LA/LC/PH | See attached |

Protocol amendments will be submitted to the Sponsor for approval prior to submission to the REC committee.
