# Supplementary material for: The Oxford study of Calcium channel Antagonism, Cognition, Mood instability and Sleep (OxCaMS): study protocol for a randomised controlled, experimental medicine study
Source: Trials. 2019 Feb 12;20:120. doi: 10.1186/s13063-019-3175-0 (PMC6373140; doi:10.1186/s13063-019-3175-0)
Supplement: Supplementary file 3 — Ethical approval letter. (DOCX 846 kb) [file 13063_2019_3175_MOESM3_ESM.docx]

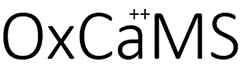


**Exploration of the short-term psychological and physical effects of calcium channel antagonism**

**on cognition and sleep.**

**Chief Investigator: Professor Paul Harrison**

**CONSENT FORM**

**/**

| **Trial ID numbers:** |  |
| --- | --- |

Please initial box

1. I confirm that I have read the Participant Information Sheet dated 2**1/12/2017**

**(version 2)** for the above study. I have had the opportunity to consider the information,

ask questions and have had these answered satisfactorily.

1. I understand that my participation is voluntary and that I am free to tell the researcher

I want to withdraw at any time, without giving any reason, and without any adverse

consequences to myself.

1. I understand that data collected during the study may be looked at by individuals from the

University of Oxford, from regulatory authorities or from the Oxford Health NHS

Foundation Trust, where it is relevant to my taking part in this research. I give permission

for these individuals to have access to my records.

1. I agree to my General Practitioner being informed of my participation in the study.
2. I confirm that I have received contact details for the research team and other specialist

services and am able to speak with them if I have any concerns or complaints.

1. I agree to researchers taking and storing and analysing my blood samples as described

in the information sheet. This will include investigation of genetic factors related to

calcium signalling and circadian rhythms.

1. I consider these samples a gift to the University of Oxford and understand that I will not

gain any direct personal benefit from this.

1. I understand that this is a research scan that is not useful for medical diagnosis, and

that scans are not routinely looked at by a doctor. If a concern is raised about a possible

abnormality on my scan, I will only be informed if a doctor thinks it is medically important

such that the finding has clear implications for my current or future health. I agree that if

abnormal activity is observed in my MEG/MRI data, this data and my identifying

details may be viewed by clinical specialists and a MEG/MRI expert.

1. I agree to a clinical neurologist contacting me to discuss medical concerns.
2. I understand that my identifying details will be stored securely and that my

permission will be sought before informing my GP of any findings.

1. I understand that the information collected about me may be used in an

anonymous form to support other research in the future. It will not be possible for

me to be identified by it.

1. I agree to take part in the study.
2. (Optional) *I agree for my anonymised samples to be used in future research,*

*here or abroad, which has ethics approval.*

**Name of Participant Date Signature**

**Name of Person taking consent Date Signature**

When completed: 1 for participant; 1 for researcher site file.

**Contact details**

The OxCaMS Team

University of Oxford Department of Psychiatry Phone: 01865 902135

Warneford Hospital Fax: 01865 223900

Oxford, OX3 7JX Email: OxCaMS@psych.ox.ac.uk
